# Spherical Rotation Dimension Reduction with Geometric Loss Functions

**Published:** 2024

**Authors:** Hengrui Luo, Jeremy E. Purvis, Didong Li

**Affiliations:** Lawrence Berkeley National Laboratory Berkeley, CA, 94720, USA; Department of Statistics, Rice University Houston, TX, 77005, USA; Department of Genetics, University of North Carolina at Chapel Hill, Chapel Hill, NC 27599, USA; Department of Biostatistics, University of North Carolina at Chapel Hill, Chapel Hill, NC 27599, USA

**Keywords:** Principal component analysis, high-dimensional data, dimension reduction

## Abstract

Modern datasets often exhibit high dimensionality, yet the data reside in low-dimensional manifolds that can reveal underlying geometric structures critical for data analysis. A prime example of such a dataset is a collection of cell cycle measurements, where the inherently cyclical nature of the process can be represented as a circle or sphere. Motivated by the need to analyze these types of datasets, we propose a nonlinear dimension reduction method, Spherical Rotation Component Analysis (SRCA), that incorporates geometric information to better approximate low-dimensional manifolds. SRCA is a versatile method designed to work in both high-dimensional and small sample size settings. By employing spheres or ellipsoids, SRCA provides a low-rank spherical representation of the data with general theoretic guarantees, effectively retaining the geometric structure of the dataset during dimensionality reduction. A comprehensive simulation study, along with a successful application to human cell cycle data, further highlights the advantages of SRCA compared to state-of-the-art alternatives, demonstrating its superior performance in approximating the manifold while preserving inherent geometric structures.

## 1. Introduction

Modern data analysis presents the challenge of high-dimensionality, where the dataset usually comes as high dimensional vectors in $R^{d}$, with a large d. Dimension reduction (DR) methods seek low dimensional representation of high dimension data (Mukhopadhyay et al., 2020; Zhang et al., 2021) to facilitate data visualization, subsequent data exploration, and statistical modeling in machine learning (Jolliffe and Cadima, 2016). Along with the difficulty in visualizations and computation, non-linearity obstructs conventional dimension reduction methods.

### 1.1 Motivation: Human Cell Cycle

In traditional DR methods (e.g., Principal Component Analysis (PCA), Pearson (1901)), it has been repeatedly pointed out that normalization preprocessing, including translations (by mean) and scalings (by standard deviation), is crucial in practicing DR (Jolliffe, 1995). However, rotation as a preprocessing step is less studied in the DR context. We are motivated by preserving non-trivial geometrical structure in DR tasks, and observed that *rotations* are as important as translations and scalings if we want to design DR methods that respect the underlying structure.

A compelling example that illustrates the need for advanced dimension reduction methods respecting the underlying structure is the analysis of cell cycle data. The cell cycle is an inherently cyclical process (Schafer, 1998) that consists of four proliferative phases: G1, S, G2, and M. Fluctuations in cell cycle genes and proteins show periodic, non-linear trends, that can be represented as a circle or sphere in a lower-dimensional space. Traditional linear methods may not adequately capture these properties, leading to the loss of crucial information.

**Figure 1:**
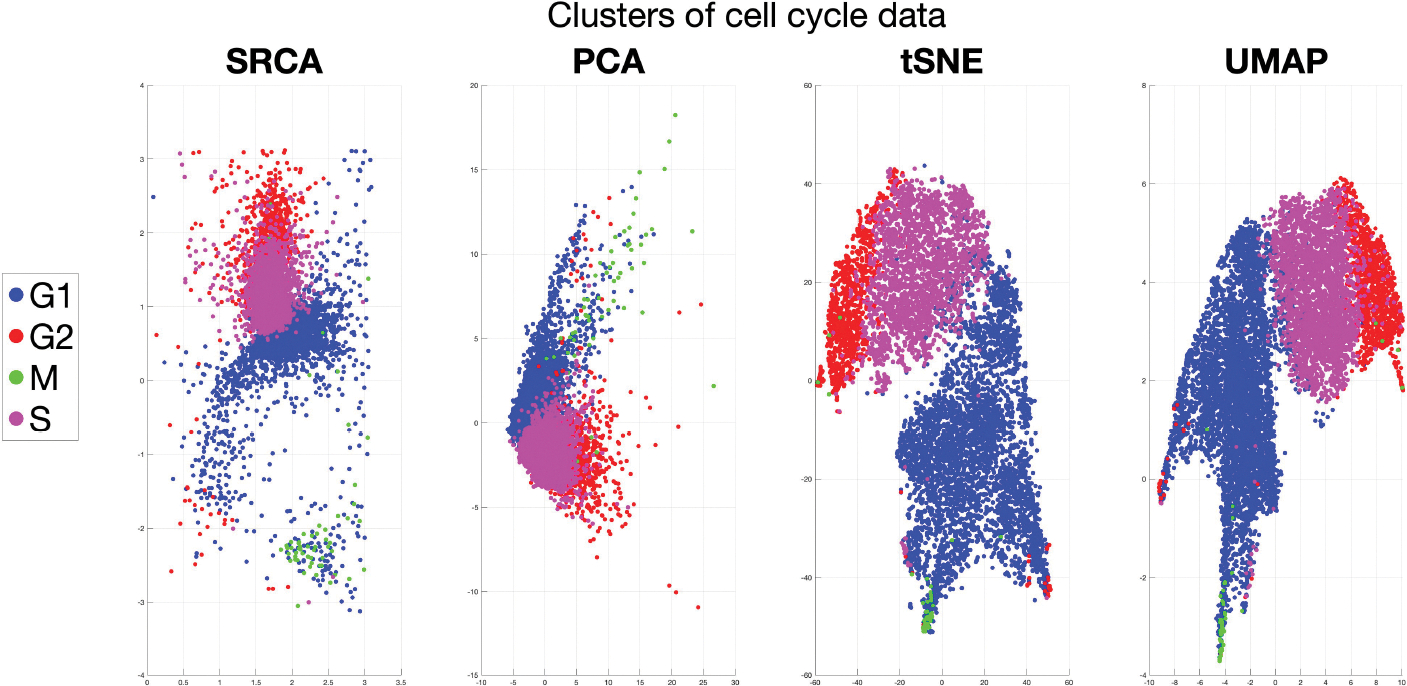
2-dimensional representation of cell cycle data, colored by different cell phases.

Figure 1 presents a 2-dimensional representation of cell cycle data proposed in Stallaert et al. (2022a), which included 40 single-cell features such as the expression or localization of core cell cycle regulators and signaling proteins. These features combine to form a multivariate cell cycle signature for each cell in the entire population, collected from 8,850 individual cells. Because individual cells are naturally asynchronous during data collection, the cells are randomly sampled over the entire cyclical distribution of possible cell cycle states. The phase of each cell (G1, S, G2, or M) was assigned using its unique molecular profile. Based on the known sequence of cell cycle phases, we would expect consecutive phases, such as G2 (red) and M (green) to be neighbors in the low dimensional projection. However, existing methods such as PCA, t-distributed Stochastic Neighbor Embedding (tSNE, Van der Maaten and Hinton (2008)), and Uniform Manifold Approximation and Projection (UMAP, McInnes et al. (2018)) (selected from the best results among other methods attempted) fail to preserve this structure in their representations.

This example motivates the development of a new DR method that utilizes spheres to represent high-dimensional data in low-dimensional spaces, effectively preserving the geometric structure and inherent cyclical nature of biological processes. In contrast to other DR methods, our proposed method, provides a representation on a 2-dimensional sphere, represented by longitude and latitude in the first panel (see Section 4.4 for more details). This SRCA representation in the lower dimensional space clearly preserves the cell cycle progression: G1→S→G2→M→G1, where the latitude (y-axis) is in the mod 2π sense, meaning π=−π. This biological periodicity, or in other words sphericity, is of central importance in analyzing cell data (Stallaert et al., 2022a). In general, disruption of these low-dimensional structures in a high-dimensional dataset (Luo et al., 2023; Luo and Strait, 2022) diminishes the effectiveness of the subsequent analysis procedure like clustering and classification.

### 1.2 Related Literature

Sphericity induced by periodicity in the above data example requires the development of sophisticated non-linear DR methods designed to preserve certain structures in the data. The common assumption is that the observetaions $x_{1},\cdots ,x_{n}$ are near a manifold M embedded in $R^{d}$. For instance, we can reformulate PCA as an optimization problem where the goal is to minimize the sum of squared distances between the original data points $x_{i}$ and their projections ${\overset{ˆ}{x}}_{i}$ onto a dimensionally reduced $d^{\prime }$-dimensional plane P. The objective function for PCA can be expressed as:

$$\underset{{\overset{ˆ}{x}}_{i}\in P\subset R^{d^{\prime }}}{min}\;\sum_{i=1}^{n}{\left\|x_{i}-{\overset{ˆ}{x}}_{i}\right\|}_{2}^{2}$$

The solution to this optimization problem seeks the best $d^{\prime }$-dimensional linear subspace P that approximates the data in the sense that P minimizes the overall point-to-plane distance. Following the reformulation of PCA as a (non-linear) optimization problem above, we can generalize to manifold families instead of the linear subspaces. In the context of dimensionality reduction, this means finding a $d^{\prime }$-dimensional manifold M within the higher-dimensional space $R^{d}$ that best captures the intrinsic geometry of the data within this specific manifold family. The objective function becomes:

$$\underset{{\overset{ˆ}{x}}_{i}\in M\subset R^{d^{\prime }}}{min}\;\sum_{i=1}^{n}d{\left(x_{i},{\overset{ˆ}{x}}_{i}\right)}^{2}$$

where $d\left(x_{i},{\overset{ˆ}{x}}_{i}\right)$ denotes the distance between $x_{i}$ and its projection ${\overset{ˆ}{x}}_{i}$ on the manifold M. We follow this generalization and consider the family of spheres in the current paper, which preserves the periodicity in the data.

Table 1 provides a selected collection of dimension reductions methods loosely categorized in two ways. Algorithms in the first row are known as “manifold learning” (Lin and Zha, 2008), which output some low dimensional features in a new Euclidean space of dimension $d^{\prime }$ instead of an estimate of M, denoted by $\hat{M}$. These methods include Locally Linear Embedding (LLE, Roweis and Saul (2000)), tSNE, , UMAP, Multi-Dimensional Scaling (MDS, Kruskal (1978)), Isomap (Tenenbaum et al., 2000), Gaussian Processes Latent Variable Model (GPLVM, Titsias and Lawrence (2010)), etc.

In contrast, the other type of DR methods, known as “manifold estimation”, which estimates M in $R^{d}$ directly, has been attracting researchers’ attention (Genovese et al., 2012). There is an immense literature in local methods including Principal Curves (PCurv, Hastie and Stuetzle (1989)), Geometric Multi-Resolution Analysis (GMRA, Allard et al. (2012)), Local Polynomial Estimator (Aamari and Levrard (2019)), Structure-Adaptive Manifold Estimation (SAME, Puchkin and Spokoiny (2022)), Spherelets (Li et al., 2022), etc. The common idea behind these methods is to partition the space into local regions, and apply local, often linear, method to each small region. The intuition is that a manifold can be locally approximated by its tangent spaces. However, these local, nonparametric, complex methods are often computationally expensive and lack of interpretability.

A recent attempt to develop a spherical analogue of PCA is Li et al. (2022), which allows us to conduct dimension reduction and learn the shape of spherically distributed datasets. However, both PCA and SPCA fail when the sample size $n<d^{\prime }$, the retained dimension (i.e., the dimension of the reduced dataset, the formal definition is introduced below) and are not easily applicable to high dimensional datasets. For instance, in the gene expression data, d is the number of genes, often over 20,000 and the retained dimension $d^{\prime }$ is often chosen to be a couple of hundreds with the largest variability (Townes et al., 2019). While the sample size could be much smaller, for example, less than 20 for certain tissues in the Genotype-Tissue Expression (GTEx) dataset (Consortium, 2020). In fact, most existing dimension reduction methods cannot handle $n<d^{\prime }$ without substantial modifications.

In this paper, we focus on parametric global methods and derive an DR method called *spherical rotation dimension reduction* (SRCA), that preserves the sphericity constraints of the dataset. Unlike some competitors, this method is applicable to high-dimensional datasets regardless of retained dimensions and sample sizes. SRCA is scalable and interpretable, and will not destroy not only the geometry but also the topology of the dataset. (see Supplement A for synthetic examples).

Specifically, we focus on biological and genetic datasets, where dimension reduction is adopted by biologist directly before clustering and subsequent tasks (Johnson et al., 2022; Zhou and Sharpee, 2018). Our method also echoes and exemplifies a grander community belief that any dimension reduction should be guided by the need of its subsequent analyses and respect the structure in the original dataset.

### 1.3 Main contributions

Our manuscript presents several significant contributions that set it apart from the existing DR methods.

SRCA introduces a novel non-linear dimension reduction technique that prioritizes interpretability in its low-dimensional representations. Notably, SRCA distinguishes itself through the direct minimization of geometric loss, providing a more intuitive approach compared to SPCA’s reliance on algebraic loss. This methodological innovation ensures that SRCA’s bias remains lower or equal to that of SPCA, enhancing the accuracy of dimensional reduction. Furthermore, SRCA’s applicability extends to scenarios where the sample size $n<d^{\prime }$, showcasing its versatility and effectiveness in handling a wide range of datasets.

On the application front, our approach not only offers fresh insights into the cyclic nature inherent in such biological processes but also represents the first instance of applying spherical DR methods in this context. The ability of SRCA to reveal biologically interpretable structures within cell cycle data marks a significant departure from previous methods like tSNE or UMAP, which, despite their utility, fall short in terms of biological interpretability for cell cycle data. Moreover, the potential of SRCA extends beyond cell cycle analysis, with implications for various biological phenomena characterized by cyclical data, such as circadian rhythms (Hogenesch and Ueda, 2011) and hormonal oscillations Kubota et al. (2012).

## 2. Methodology

In this section, we outline the proposed procedure that aims to minimize a geometric loss function, specifically the mean squared errors between the original data points $x_{i}$ and dimension-reduced data points $\hat{x_{i}}$.

We denote the intrinsic dimension of the support of the reduced dataset by $d^{\prime }$ and refer to it as the *retained dimension*. It is worth noting that some literature uses the embedded dimension as $d^{\prime }$. For example, if the reduced dataset lies in $S^{1}$ embedded into $R^{2}$, we would consider the dimensionality of the reduced data to be $d^{\prime }=1$ and not 2, as $S^{1}$ is a one-dimensional manifold.

### 2.1 PCA and SPCA Revisited

As discussed in Section 1.2, PCA identifies a low-rank linear subspace from observations $𝒳=x_{1},\cdots ,x_{n}\subset R^{d}$ by minimizing the sum of squared error loss function:

$$\underset{V\in R^{d\times d^{\prime }}}{min}\;\sum_{i=1}^{n}{\left\|x_{i}-\hat{x_{i}}\right\|}^{2}=\sum_{i=1}^{n}{\left\|x_{i}-\overset{‾}{x}-VV^{T}\left(x_{i}-\overset{‾}{x}\right)\right\|}^{2},\;\text{s.t.}\;V^{T}V=I_{d^{\prime }}.$$

where $\overset{‾}{x}=\frac{1}{n}\sum_{i=1}^{n}x_{i}$ is the sample mean calculated in $R^{d}$. The solution to this optimization problem yields a rotation matrix V that defines a subspace, called the solution to PCA.

From an optimization perspective, PCA is a problem for a given (geometric) loss function (Journée et al., 2010), which quantifies the $l_{2}$ errors between the observation and the subspace. SPCA aims to find the optimal sphere S. Unlike PCA’s planar solution, the solution of SPCA is a sphere with center c and radius r residing in the linear subspace V to fit the data. SPCA does not minimize the sum of squared distances between observations and the sphere; instead, it employs a two-step algorithm to minimize the sum of point-to-plane and projection-to-sphere distances.

The desired one-step algorithm was not explored in the original paper (Li et al., 2022) since the problem is theoretically more complicated and lacks a closed-form solution (See Supplement G). In contrast, our proposed SRCA can be shown to attain this one-step goal.

### 2.2 Geometric Loss Function

Given a sphere centered at c with radius r, we first assume that it lies in a subspace parallel to a coordinate plane in $R^{d}$ determined by ℐ⊂{1,⋯,d} after a linear transformation determined by a (non-singular) matrix W. We denote such low-dimensional “sub-sphere” by $S_{ℐ}(c,r)$ and use the notation $I_{ℐ}$ to denote an identity matrix with ones in (i,i)-th entries, i∈ℐ but zeros in the rest entries, then the point-to-sphere distance from a generic point $x_{i}$ to this sphere can be expressed as

$$\begin{array}{l} d{\left(x_{i},S_{ℐ}(c,r)\right)}^{2}\;={\left(x_{i}-c\right)}^{T}WI_{{ℐ}^{c}}\left(x_{i}-c\right)+{\left(\sqrt{{\left(x_{i}-c\right)}^{T}{\sqrt{W}}^{T}I_{ℐ}\sqrt{W}\left(x_{i}-c\right)}-r\right)}^{2}\\ \;={\left(x_{i}-c\right)}^{T}W\left(x_{i}-c\right)+r^{2}-2r\sqrt{{\left(x_{i}-c\right)}^{T}{\sqrt{W}}^{T}I_{ℐ}\sqrt{W}\left(x_{i}-c\right).} \end{array}$$

**Figure 2:**
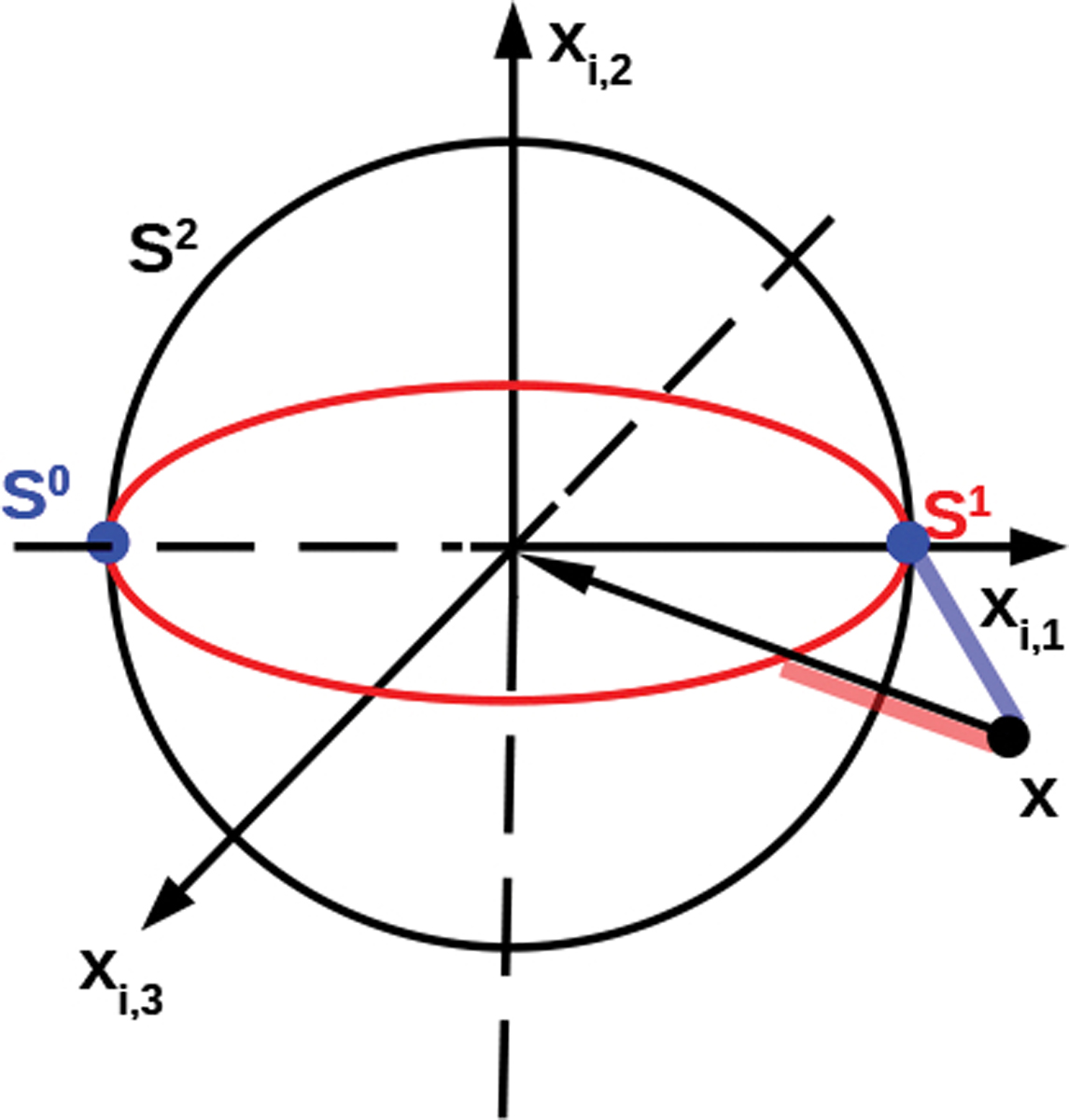
Illustration of dimension reduction via a sphere. The black point x is projected onto the one-dimensional (red circle, $S^{1}$) or zero-dimensional (blue points, $S^{0}$) in the ambient space $R^{3}$. The transparent line segments shows the corresponding point-to-sphere distances.

Assume W=I, the Figure 2 illustrates a $R^{3}$ space where a two-dimensional sphere $S^{2}$ is embedded, and we wish to reduce our data onto a one-dimensional (a circle $S^{1}$) or zero-dimensional (points $S^{0}$) sphere. The $S^{0}$ and $S^{1}$ are defined by ℐ and the sphere’s center c and radius r. In Li et al. (2022), the distance from the point x to the sphere was decomposed into two components: the Euclidean distance to the subspace (along the axis $x_{i,3}$) and the distance within this subspace (spanned by the axes $x_{i,1}$ and $x_{i,2}$) to the sphere, which were optimized separately. In the current paper, we optimize this distance in one step and hence obtain better solution than that from Li et al. (2022). Then, Equation (1) generalizes from this observation by incorporating a weight matrix W.

When W≠I, the interpretation of the distance measure would change. It would no longer represent the point-to-sphere distance but rather a weighted distance where the contribution of each dimension is scaled according to W. In such a case, the optimization problem would aim to find the best-fitting sphere in this anisotropic space defined by W. The matrix W in PCA-like DR (See Section 1.2) can prioritize features through weight assignment, inversely proportional to their variance, akin to PCA normalization. Positive definiteness of W leads to Mahalanobis distances, aligning data scaling and inter-feature correlations, mirroring PCA’s covariance adjustment (Scheffler et al., 2020). In this situation, the matrix W transforms the sphere to lies in a coordinate plane so that the point-to-sphere distance admits a closed form. With this point-to-sphere distance, the (geometric loss) function can be written as

$$\begin{array}{l} ℒ(c,r,ℐ|𝒳,W)\;=\sum_{i=1}^{n}\;\left({\left(x_{i}-c\right)}^{T}W\left(x_{i}-c\right)+r^{2}-2r\sqrt{{\left(x_{i}-c\right)}^{T}{\sqrt{W}}^{T}I_{ℐ}\sqrt{W}\left(x_{i}-c\right)}\right)\\ \;=:\sum_{i=1}^{n}\;\rho \left(x_{i};\theta \right), \end{array}$$

where we use the notation θ=(c,r) with a given ℐ, and the notation $\rho \left(x_{i};\theta \right)$ is adopted to emphasize its additive form and to facilitate the later theoretic discussions. Our dimension reduction procedure can be described as solving the optimization problem below:

(1) $$\underset{ℐ\subset \{1,\cdots ,d\},c\in R^{d},r\in R^{+}}{min}\;ℒ(c,r,ℐ|𝒳,W)=\underset{ℐ\subset \{1,\cdots ,d\},c\in R^{d},r\in R^{+}}{min}\;\sum_{i=1}^{n}\;\left({\left(x_{i}-c\right)}^{T}W\left(x_{i}-c\right)+r^{2}\right.\;\left.\;-2r\sqrt{{\left(x_{i}-c\right)}^{T}{\sqrt{W}}^{T}I_{ℐ}\sqrt{W}\left(x_{i}-c\right)}\right),\text{s.t.}|ℐ|=d^{\prime }+1$$

Using the loss function defined in (1), we can simultaneously estimate the center c, radius r and ℐ by solving the optimization problem. Since there are at most $2^{d}$ possible choices of ℐ, it is straightforward to verify that this one-step optimization problem (1) can be equivalently solved in a two-step procedure: First, select a subset of indices ℐ⊂{1,⋯,d}, and then estimate the center c and radius r of the dataset 𝒳. This optimization problem can also be solved iteratively on variables ℐ,r,c. The binary search can be the first step, followed by esimating the center c and radius r:

1. Given c and r, perform an exhaustive binary search among all possible ℐ.
2. Given ℐ and c, take the derivative of $\frac{\partial ℒ}{\partial r}=0$ to obtain:
$$r=\frac{1}{n}\sum_{i=1}^{n}\sqrt{{\left(x_{i}-c\right)}^{T}{\sqrt{W}}^{T}I_{ℐ}\sqrt{W}\left(x_{i}-c\right)}.$$
3. Given ℐ and r, take the derivative of $\frac{\partial ℒ}{\partial c}=0$ to obtain:
$$\frac{\partial ℒ}{\partial c}=\sum_{i=1}^{n}\left(-2W\left(x_{i}\;-\;c\right)+2r\frac{I_{ℐ}\sqrt{W}\left(x_{i}\;-\;c\right)}{\sqrt{{\left(x_{i}\;-\;c\right)}^{T}{\sqrt{W}}^{T}I_{ℐ}\sqrt{W}\left(x_{i}\;-\;c\right)}}\right)=0.$$

Observe that if j∈ℐ, the j-th coordinate of the second term is zero, so we have $c_{j}=\frac{1}{n}\sum_{i=1}^{n}x_{i,j}$ for any $j\in {ℐ}^{c}$. For j∈ℐ, an analytic solution is difficult to find, but gradient descent can provide a numerical solution.

So far, we have assumed that the underlying support $S_{ℐ}$ has coordinate axes that are parallel to the coordinate axes. To make this assumption more realistic, we propose to rotate the dataset 𝒳 so that it can be viewed in a position such that its axes are parallel to the coordinate axes. Then, we solve the optimization problem (1) for the rotated dataset and rotate it back to obtain reduced dataset. The rotation can be chosen according to the types of datasets as appropriate in the procedure, as is discussed in Sec.4.5.

The exhaustive binary search over $2^{d}$ possible subsets is computationally expensive. We observe that the core of the optimization problem lies in the subset selection of index set {1,⋯,d}. We can rephrase the optimization problem (1) as follows:

(2) $$\begin{array}{l} \underset{c\in R^{d},r\in R^{+}}{min}\;\sum_{i=1}^{n}({\left(x_{i}-c\right)}^{T}W\left(x_{i}-c\right)+r^{2}\\ \;\left.-2r\sqrt{{\left(x_{i}-c\right)}^{T}{\sqrt{W}}^{T}v^{T}Iv\sqrt{W}\left(x_{i}-c\right)}\right)\;,\text{s.t.}\|v{\|}_{l_{0}}=d^{\prime }+1, \end{array}$$

Since $l_{0}$-norm is not convex, solving this problem requires a brute-force step in finding optimal v whose entries are either 0 or 1 (and hence ℐ since $v^{T}Iv=I_{ℐ}$), as detailed in Algorithm 2. Instead of using $l_{0}$ directly in the original problem, we consider the following computationally cheaper alternative:

(3) $$\begin{array}{l} \;\underset{c\in R^{d},r\in R^{+}}{min}\;\sum_{i=1}^{n}\;\left({\left(x_{i}-c\right)}^{T}W\left(x_{i}-c\right)+r^{2}-2r\sqrt{{\left(x_{i}-c\right)}^{T}{\sqrt{W}}^{T}v^{T}Iv\sqrt{W}\left(x_{i}-c\right)}\right),\\ \;\text{s.t.}\;\|v{\|}_{l_{1}}\leq d^{\prime }+1, \end{array}$$

where $l_{1}$ norm is used as a convex surrogate norm. This kind of relaxation is proposed in optimization (Boyd et al., 2004). As a practical suggestion, when there are more than 500 combinations of binary indices to search exhaustively, we recommend $l_{1}$ relaxation as a more scalable solution in (3), otherwise we perform an exhaustively search. For very high-dimensional dataset, the empirical performances for $l_{0}$ and $l_{1}$ penalties are similar.

### 2.3 SRCA Method

The method discussed above is refereed to as the *spherical rotation dimension reduction* (SRCA) method and presented in Algorithm 1, which employs geometric loss functions designed for spherical datasets. The key steps of our proposed SRCA dimension reduction method can be summarized into a “Rotate-Optimize-Project” scheme as follows, with $l_{1}$ algorithms detailed in Supplement C and branch-and-bound implementation in Supplement J.

#### Rotate: Conduct the rotation.

With the chosen rotation method, we construct a rotation matrix R based on the dataset 𝒳. We translate and rotate the dataset 𝒳 to a standard position $(𝒳-\bar{𝒳})R$, so that we can reasonably assume that the axes of the ellipsoid are parallel to the coordinate axes (Jolliffe, 1995).


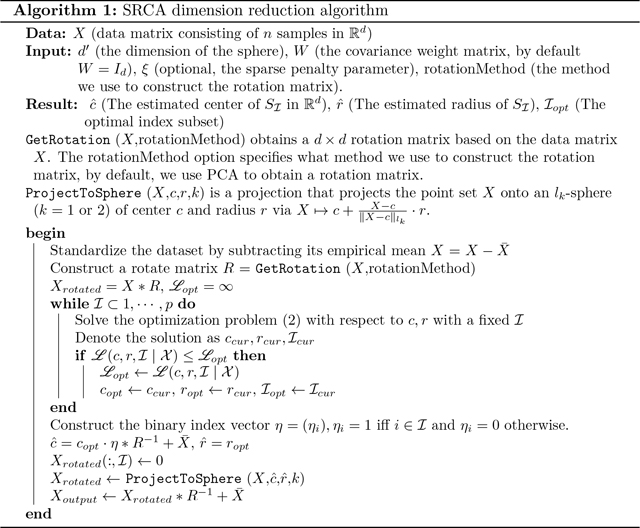


#### Optimize: Solve the optimization for the best $d^{\prime }+1$ axes.

We perform dimension reduction based on the geometric loss function discussed above. As stated in (2), we conduct dimension reduction by minimizing the loss function based on the point-to-sphere distance to the estimated sphere $S_{ℐ}$, to obtain the optimal $v_{\text{opt}}$ and the optimal index set ${ℐ}_{\text{opt}}$.

(4) $$\begin{array}{l} \left(v_{opt},c_{opt},r_{opt}\right)=arg\underset{c\in R^{d},r\in R^{+}}{min}\;\sum_{i=1}^{n}\;({\left(x_{i}-c\right)}^{T}W\left(x_{i}-c\right)+r^{2}\\ \;\left.-2r\sqrt{{\left(x_{i}-c\right)}^{T}{\sqrt{W}}^{T}v^{T}Iv\sqrt{W}\left(x_{i}-c\right)}\right)\text{s.t.}\|v{\|}_{l_{0}}=d^{\prime }+1, \end{array}$$

where the constraint can be relaxed by $\|v{\|}_{l_{1}}\leq d^{\prime }+1$.

#### Project: project onto the optimal sphere.

Now we project the datapoints back into full space with the chosen dimension and axes, placing $x_{i}$ back onto the sphere $S\left(c_{\text{opt}},r_{\text{opt}}\right)$ with the estimated center $c_{\text{opt}}$ and radius $r_{\text{opt}}$, the SRCA projection is given by:

(5) $${\hat{x}}_{i}=c_{opt}\cdot v_{opt}+r_{opt}\frac{\left(x_{i}\;-\;c_{opt}\right)v_{opt}^{T}Iv_{opt}}{\left\|\left(x_{i}\;-\;c_{opt}\right)v_{opt}^{T}Iv_{opt}\right\|}.$$

A natural extension of SRCA is to incorporate the rotation matrix R as a parameter to be optimized, which may further enhance model adaptability. However, this will introduce a challenging high-dimensional optimization problem. Unlike optimizing for center c and radius r, which is a lower-dimensional optimization problem completed by binary search, optimizing a $d^{\prime }$-dimensional symmetric matrix, especially without sparsity constraints, becomes more complex and computationally intensive.

## 3. Theoretical Results

We have established the procedure for our propose method, SRCA, in an algorithmic way. Next, we discuss and provide some theoretical results that guarantee the performance of SRCA in applications. Proofs are deferred to supplementary materials, but we want to emphasize that the techniques of ρ-loss (Huber et al., 1967) and Γ-convergence (Braides et al., 2002) are introduced to tackle probabilistic properties for DR methods.

### 3.1 Convergence

Unlike SPCA, SRCA does not have a closed form solution (i.e., analytic expression of center and radius estimates in terms of dataset 𝒳) but relies on the solution to an optimization problem. Therefore, the convergence of this optimization becomes central in our theory development. We briefly discuss the convergence guarantee for the algorithm we designed. In the binary search situation, for each fixed choice of indices, we compute the gradient of loss function. With mild assumptions, gradient descent provides linear convergence. If the optimization problem (4) has solutions, then the solution is clearly unique. This is because there are only finitely many v such that $\|v{\|}_{l_{0}}=d^{\prime }+1$, and the binary search in the standard algorithm would exhaustively search all possible values of v.

To these ends, we provide a basic convergence for a sub-problem in our Algorithm 1 (without $l_{1}$ penalty) via the gradient descent algorithm of positive constant step size. The sub-problem is defined by the following loss function:

(6) $${ℒ}_{v}(c,r|𝒳,W)={ℒ}_{v}(c,r):=\;\sum_{i=1}^{n}\;\left({\left(x_{i}-c\right)}^{T}W\left(x_{i}-c\right)+r^{2}\right.$$

(7) $$\left.-2r\sqrt{{\left(x_{i}-c\right)}^{T}{\sqrt{W}}^{T}v^{T}Iv\sqrt{W}\left(x_{i}-c\right)}\right)$$

The following theorem guarantees the convergence of SRCA.

#### Theorem 1

For a fixed vector v (or equivalently $I_{ℐ}$), if we assume that $\left\|x_{i}-c\right\|\leq R_{1},\;\forall i=1\cdots ,n,\;r\leq R_{2}$ and $\left|\lambda_{max}(W)\right|\leq R_{3}$ for positive constants $R_{1},\;R_{2},\;R_{3}$, where $\lambda_{max}(\cdot )$ denotes the largest eigenvalue, then for a positive finite constant step size independent of the iteration number k, the gradient descent algorithm (c.f., the setting in Boyd et al. (2003, 2004)) converges to the optimal value in the following sense,

$$\underset{k\to \infty }{lim}\;{ℒ}_{v,k}\to {ℒ}_{v}^{*}$$

where ${ℒ}_{v,k}={ℒ}_{v}\left(c_{k},r_{k}\right),\left(c_{k},r_{k}\right)$ is the value in the k-th iterative step in the gradient descent algorithm, and ${ℒ}_{v}^{*}$ denotes the minimum of the loss function for this fixed v.

These results justify that for a fixed v (or equivalently ℐ) we can solve the sub-problem defined by the above function, and since we conduct an exhaustive search for the index vector v, we can find the solution to the original problem (4) as well.

### 3.2 Consistency

In this section, we assume the observed data are from a “true” but unknown sphere $S_{I_{0}}\left(c_{0},r_{0}\right)$ and show that the solution of SRCA is consistent, that it, we can find the true sphere as long as we have enough samples.

#### Theorem 2

Assume $x_{i}\in S_{{ℐ}_{0}}\left(c_{0},r_{0}\right),\forall i=1,\cdots ,n$ and $n>d^{\prime }+1$. Let ${\hat{ℐ}}_{k}$, ${\hat{c}}_{k}$, ${\hat{r}}_{k}$ be the solution of SRCA after k iteration in the solution of the corresponding optimization problem, then

$$({\hat{ℐ}}_{k},{\hat{c}}_{k},{\hat{r}}_{k})\overset{k\to \infty \;}{\to }\left({ℐ}_{0},c_{0},r_{0}\right).$$

However, the assumption that are observations are exactly on a sphere is unrealistic in practice, as the data often come with measurement errors. Instead, we adopt following (common) assumption in manifold estimation: $x_{i}=y_{i}+\epsilon_{i}$, where the unobserved $y_{i}$’s are exactly from a sphere $S\left(c_{0},r_{0}\right)$ and $\epsilon_{i}$ represents the measurement error. The next theorem fills in the gap using the Γ-convergence (Braides et al., 2002), which is first applied in DR problems.

#### Theorem 3

Under the following assumptions:

(A0) The index vector ℐ is fixed and the parameter $\theta =(c,r)\in \Theta :=[-C,C{]}^{d}\times \left[R_{0},R\right]\subset R^{d}\times R^{+}$ for some $C,R_{0},R>0$.

(A1) $x_{i}$ ’s are compactly supported.

(A2) $\lim_{n\to \infty }\;\frac{1}{n}\sum_{i=1}^{n}\;\left\|\epsilon_{i}\right\|=0$

the SRCA solution ${\overset{ˆ}{\theta }}_{n}\to \theta_{0}$ as n→∞.

In other words, the SRCA estimator based on noisy samples is consistent, that is, converges to the true parameter $\theta_{0}$, as low as the noise decays to zero with sample size. In fact, this assumption is even weaker than those in existing literature, see Maggioni et al. (2016); Fefferman et al. (2018); Aamari and Levrard (2019) for more details. For example, in Aamari and Levrard (2019) the amplitude of the noise is assume to be $\|\epsilon \|∼n^{-\frac{\alpha }{d}}$ for α>1. In contrast, we only require ‖ϵ‖→0, so $\|\epsilon \|∼n^{-\alpha }$ for any α>0 or even $\|\epsilon \|∼\frac{1}{log\;n}$ is good enough.

### 3.3 Asymptotics

In this section, we consider the asymptotic behavior of SRCA optimization result when the underlying dataset is assumed to be drawn from a probabilistic distribution, regardless whether it is supported on a sphere or not.

To yield the asymptotic results, we need to take the perspective of robust statistics as mentioned in the end of Section F. The asymptotic theory here is a specific case of empirical risk minimization. With a mild technical assumption that the parameter $\theta =\left(c,r\right)\in \Theta :=\;[-C,C{]}^{d}\times \left[R_{0},R\right]\subset R^{d}\times R^{+}$ for some $C,R_{0},R\in (0,\infty )$, our loss function and optimization problem can be expressed as

(8) $$\overset{min}{ℐ\subset \{1,\cdots ,d\},c\in [-C,C{]}^{d}\subset R^{d},r\in \left[R_{0},R\right]\subset R^{+}}\;ℒ(c,r,ℐ|𝒳,W)$$

(9) $$=\overset{min}{ℐ\subset \{1,\cdots ,d\},c\in [-C,C{]}^{d}\subset R^{d},r\in \left[R_{0},R\right]\subset R^{+}}\;\sum_{i=1}^{n}\;({\left(x_{i}-c\right)}^{T}W\left(x_{i}-c\right)+r^{2}\;\left.-2r\sqrt{{\left(x_{i}-c\right)}^{T}{\sqrt{W}}^{T}I_{ℐ}\sqrt{W}\left(x_{i}-c\right)}\right)\text{s.t.}|ℐ|=d^{\prime }+1$$

For a fixed ℐ, (9) can be written in the form of (3.1) in Huber (2004), i.e.,

$$\rho (x;\theta )=\left({\left(x_{i}-c\right)}^{T}W\left(x_{i}-c\right)+r^{2}-2r\sqrt{\left(x-c{)}^{T}{\sqrt{W}}^{T}I_{ℐ}\sqrt{W}(x-c\right)}\right)$$

Correspondingly, we can write Huber’s ψ-type function of ρ as $\psi (\theta )=\frac{\partial \rho (\theta )}{\partial \theta }$.

Classical style asymptotic results are presented below in Theorem 4, which states that, with mild assumptions, the estimates $T_{n}$ obtained by solving SRCA would estimate the center and radius of the spherical space consistently, corresponding to Huber’s ρ-type estimator consistency (Huber et al., 1967); Theorem 5 states that with more stringent conditions on continuity of $T_{n}$, asymptotic normality of these estimators can also be formulated into Huber’s ψ-type normality.

To apply these two asymptotic results, we need to make mild assumptions on the parameter space and assume that we already know the retained dimension $d^{\prime }+1$.

#### Theorem 4

Suppose (A0) in Theorem 3 holds and the samples $x_{1},\cdots ,x_{n}\in X=R^{d}$ of size n are i.i.d. drawn from the common distribution P. P has finite second moments on the probability space (X,𝒜,ν) with Borel algebra 𝒜 and Lebesgue measure ν. Then the consistent estimator $T_{n}$ for parameter θ=(c,r) defined by

$$\frac{1}{n}\sum_{i=1}^{n}\rho \left(x_{i};T_{n}\right)-\underset{\theta \in \Theta }{inf}\;\frac{1}{n}\sum_{i=1}^{n}\rho \left(x_{i};\theta \right)\overset{n\to \infty \;}{\to }0,\;\mathrm{a.s.}\;P$$

would converge in probability and almost surely to $\theta_{0}$ w.r.t. P (for the true parameter values $\theta_{0}$ defined on page 46). Particularly, $T_{n}$ can be realized as a solution to our optimization problem (8) above.

Unlike Theorem 1, which concerns the convergence of the algorithm, we assume that the fixed i.i.d. samples 𝒳 are drawn from a probability distribution. Similarly, we have a distributional result as follows.

#### Theorem 5

In addition to the assumption in Theorem 4, we assume $P\left(\left|T_{n}-\theta_{0}\right|\leq \eta \right)\to 1$ as n→∞, then the estimator $T_{n}$ defined by

$$\frac{1}{\sqrt{n}}\sum_{i=1}^{n}\psi \left(x_{i};T_{n}\right)\overset{n\to \infty \;}{\to }0,\;\mathrm{a.s.}\;P$$

would satisfy $\frac{1}{\sqrt{n}}\sum_{i=1}^{n}\psi \left(x_{i};T_{n}\right)+\sqrt{n}\lambda \left(T_{n}\right)\overset{n\to \infty \;}{\to }0$, a.s. P. (where λ(⋅) is defined in the (N-1) in Section H) Particularly, our loss function would satisfy differentiability at $\theta_{0}$ and $\sqrt{n}\left(T_{n}-\theta_{0}\right)$ is asymptotically normal with mean zero and covariance matrix

$$\left(\nabla_{\theta_{0}}\lambda^{-1}\right)\cdot \left({\left[\psi \left(x_{i};\theta_{0}\right)-E_{P}\psi \left(x_{i};\theta_{0}\right)\right]}^{T}\left[\psi \left(x_{i};\theta_{0}\right)-E_{P}\psi \left(x_{i};\theta_{0}\right)\right]\right)\cdot {\left(\nabla_{\theta_{0}}\lambda^{-1}\right)}^{T}.$$

Defined by the geometric loss function ℒ, SRCA does not have an analytic solution, but this loss benefits from the theoretic results above and can be replaced by other types of loss functions, enabling SRCA to be applied more widely.

Note that we also assumed that the index set ℐ is fixed for our statements of theorems. In the exhaustive search, these results above can be applied individually to fixed ℐ; but in the $l_{1}$-relaxed problem (4), since the optimization is a joint optimization our asymptotic results Theorem 4 and 5 in this section do not apply.

### 3.4 Loss Function Minimization

Next, we consider the theoretic behavior of SRCA in terms of approximating a general manifold. The following theorem compares the MSE of PCA, SRCA (when the rotation is chosen by PCA) and SPCA:

#### Theorem 6

Given data $x_{1},\cdots ,x_{n}$ in a bounded subset of $R^{d}$, let H be the best subspace obtained by PCA, $S_{1}$ be the sphere obtained by SPCA and $S_{2}$ be the sphere obtained by SRCA with rotation provided by PCA, then

$$\sum_{i=1}^{n}d^{2}\left(x_{i},S_{2}\right)\leq min\left\{\sum_{i=1}^{n}\;d^{2}\left(x_{i},H\right),\sum_{i=1}^{n}\;d^{2}\left(x_{i},S_{1}\right)\right\}.$$

That is, SRCA has the best approximation performance in terms of MSE among PCA, SPCA and SRCA, regardless of the true support of the observations.

To summarize and interpret our theoretical results briefly, Theorem 1 ensures that a gradient-descent algorithm can be used for solving the loss function minimization problem (1) for any finite samples with convergence guarantees; Theorem 2 and 3 show that SRCA can recover the true sphere, if it exists, when the data are clean or with measurement error. For general case where the observations are not necessarily supported by a sphere, Theorems 4 and 5 ensure that the sequence of finite-sample minimizers of our loss function asymptotically converges to minimizer $\theta_{0}$; Theorem 6 points out that SRCA can better approximate the unknown support in terms of MSE than PCA and SPCA.

## 4. Numerical Experiments

With theoretical results above on MSE, we also wish to examine the practical performance of SRCA against the state-of-the-art dimension reduction methods on real datasets. We focus on the empirical structure-preserving and coranking measurements below, an application to the motivating dataset about cell cycle, and discuss the choice of parameters in SRCA in the end. Details of selected datasets are in Supplement D.

### 4.1 MSE

As a dimension reduction method, the most common and natural measurement of performance is based on the mean squared error (MSE) between the original and reduced datasets, which measures how close the manifold is to the original observations. However, most dimension reduction methods only output low dimension features, like LLE, Isomap, tSNE, UMAP, GPLVM, etc, where the MSE is not well-defined because the low dimensional features cannot be trivially embedded into original data space $R^{d}$. Algorithms that output projected data in the original space $R^{d}$ include SPCA, PCA, and our proposed SRCA.

Table 2 presents the MSEs of three competing algorithms on these datasets with $d^{\prime }=min\{d-1,\;4\}$. The out-sample MSEs show a similar patterns and is postponed to the Supplement I. It is evident that SRCA has the property of MSE minimization for most datasets and most $d^{\prime }$, as predicted by the theory in Theorem 6.

### 4.2 Cluster Preserving

Cluster structure properties of different dimension reduction algorithms varies, however, we hope that the data points belonging to the same group in the original dataset, are close together in the dimension-reduced dataset.

For visualization purposes, we fix retained dimension to be $d^{\prime }=2$ and compare the following six algorithms: SRCA, SPCA, PCA, LLE, tSNE, UMAP. We choose these state-of-the-art competitors to visualize in 2-dimensional figures. For PCA, we present the first two PCs; the coordinates in LLE, tSNE and UMAP are not interpretable. For SRCA and SPCA, since the projected data are on a 2-dimensional spheres, we present the polar angle and azimuthal angle, which are within [−π,π].

To further quantify the how well the clustering structures are preserved, the Silhouette Score (SC, (Rousseeuw, 1987)), Calinski-Harabasz Index (CHI, (Caliński and Harabasz, 1974)) and Davis-Bouldin index (DBI, (Davies and Bouldin, 1979)) are considered. Higher SC and CHI, lower DBI imply better separation between clusters in the dataset. We provide these measures on the original labeled dataset (without any DR) as baselines.

**Figure 3:**
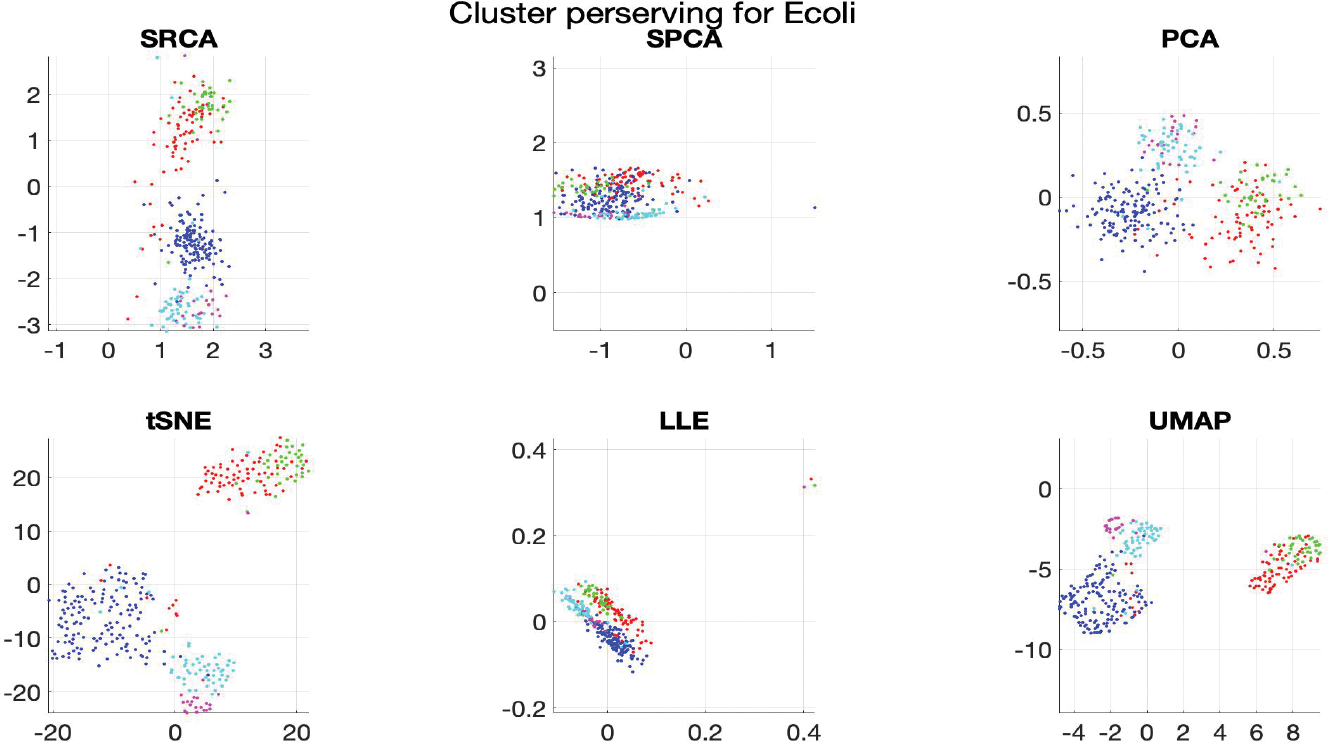
Cluster structures for Ecoli, $d^{\prime }=2$, there are five different clusters represented by different colors in the reduced dataset.

Figure 3 and Table 3 show that SRCA outperforms SPCA, PCA and LLE in terms of all three metrics and comparable to tSNE and UMAP. SRCA also has advantage over its predecessor SPCA and simpler linear method like PCA. With these experiments and Supplement B, we conclude that if the dataset has strong spatial sphericity, we usually have good cluster preserving properties from SRCA. If the dataset is highly non-linear, tSNE and UMAP are usually better at the cost of creating fake clusters if tuning parameters are not well-chosen (Wattenberg et al., 2016; Wilkinson and Luo, 2022).

### 4.3 Coranking Matrix

Another type of quantitative measures is based on the *coranking matrix* (Lee and Verleysen, 2009; Lueks et al., 2011). The coranking matrix can be viewed as the joint histogram of the ranks of original samples and the dimension-reduced samples. The coranking matrix can be used to assess results of dimension reduction methods. Entry $q_{kl}$ in the coranking matrix is defined as $q_{kl}:=\left\{(i,j)|\rho_{ij}=k\right.\;and\;\left.r_{ij}=l\right\}$, where $\rho_{ij}:=\left\{k:d\left(x_{i},x_{k}\right)<\right.\;d\left(x_{i},x_{j}\right)\;or\;d\left(x_{i},x_{k}\right)=d\left(x_{i},x_{j}\right),k<j\}$ stores the rank of the pair $x_{i},\;x_{j}$ in the original dataset; $r_{ij}:=\left\{k:d\left({\overset{ˆ}{x}}_{i},{\overset{ˆ}{x}}_{k}\right)<d\left({\overset{ˆ}{x}}_{i},{\overset{ˆ}{x}}_{j}\right)\right.$ or $\left.d\left({\overset{ˆ}{x}}_{i},{\overset{ˆ}{x}}_{k}\right)=d\left({\overset{ˆ}{x}}_{i},{\overset{ˆ}{x}}_{j}\right),k<j\right\}$ stores the rank of the pair ${\overset{ˆ}{x}}_{i},\;{\overset{ˆ}{x}}_{j}$ in the dimension-reduced dataset, where the rank pair reversed in the dimension-reduced dataset. An ideal dimension reduction method should preserve all the ranks of these pairwise distances between original and reduced datasets. That is, we have identical ordering of these pairwise distances in the original space and the dimension-reduced space. Coranking matrix is a finer summary but is related to ijk rank test (See, e.g., Solomon et al. (2021)).

We provide three scores (the higher the better) related to coranking matrices of the dimension-reduced results: CC (cophenetic correlation, measuring correlation between distance matrices), AUC (area under curve for the $R_{NX}$ score), WAUC (weighted AUC) computed from coRanking R-package (Kraemer et al., 2018).

**Figure 4:**
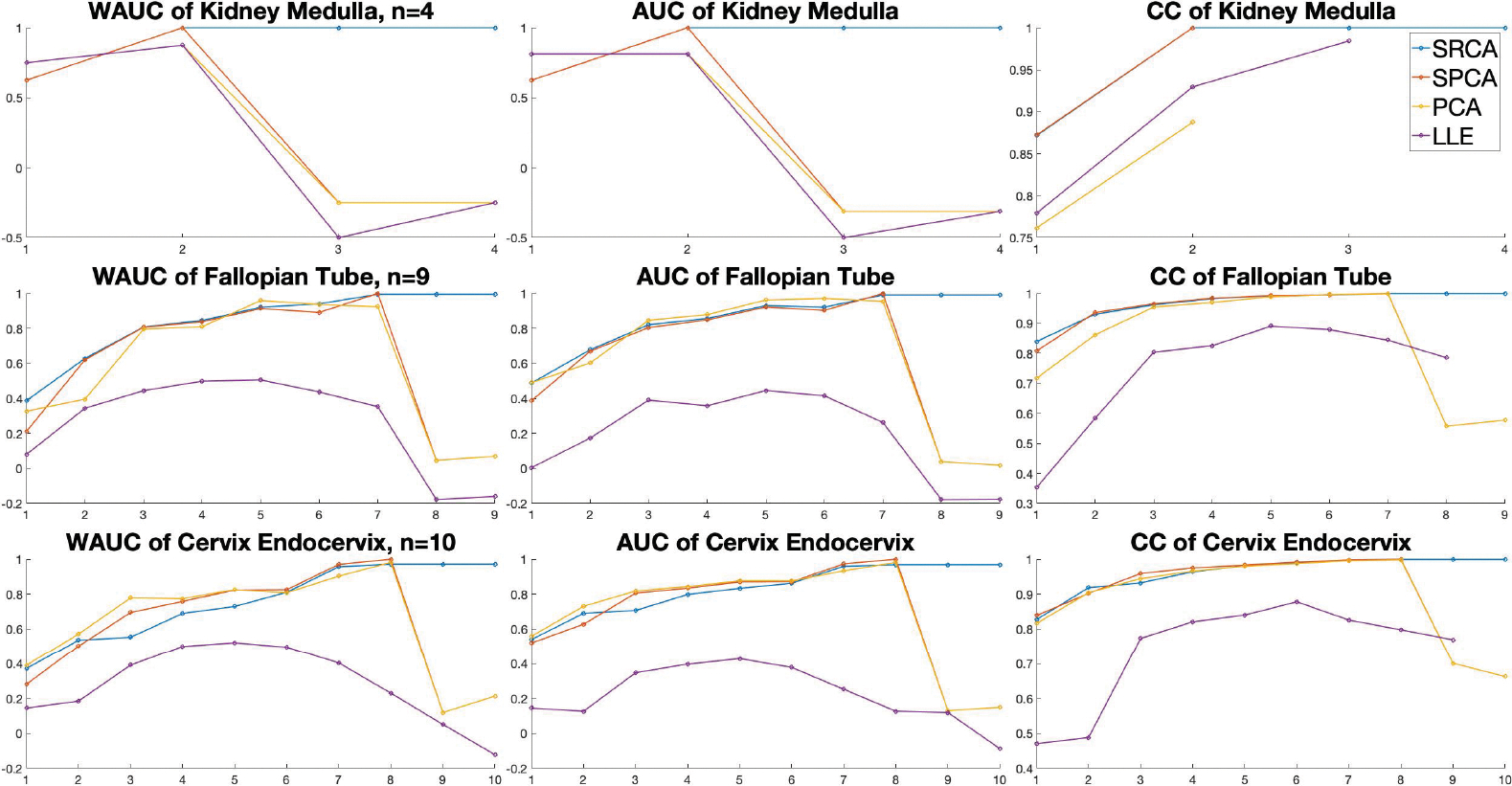
Coranking measurements of three GTEx tissues for different retained dimension, the horizontal axes are retained dimension $d^{\prime }$, the vertical axes are score values.

To understand our subsequent analyses better, we referred our readers to the analysis of the dimension reduction result of simple examples like $S^{2},\;T^{2}$ and a plane diffeomorphic to $R^{2}$, evaluated by these coranking matrix related scores in Supplement B, where SRCA is the only DR method that consistently behaves almost the best in plane, spheres and topologically non-trivial examples like torus when measured by coranking scores. Another advantage of SRCA over existing DR methods is that it allows $n<d^{\prime }$, which happens to a variety of real datasets, specially for biomedical data where both d and $d^{\prime }$ are large. For example, in Genotype-Tissue Expression(GTEx) dataset (Consortium, 2020), some tissues are hard to collect so the sample sizes are small but the dimension is very high, like Kidney Medulla (n=4), Fallopian Tube (n=9) and Cervix Endocervix (n=10). However, there are thousands of genes so we expect that the intrinsic dimension $d^{\prime }>n$. Following the common practice of feature selection in this database, we subsetted the data to the most variable 500 genes (Townes et al., 2019). Most competitors mentioned before are not directly applicable anymore when $d^{\prime }>n$, including tSNE, UMAP, Isomap, MDS, etc. For illustration purpose, we retain the first n dimensions. As a result, we present the three coranking based measurements on three tissues obtained from SRCA, SPCA, PCA and LLE for different $d^{\prime }$ in Figure 4.

### 4.4 Application: Human Cell Cycle

The human cell cycle consists of four growth phases: G1, S, G2, and M. In a recent study, non-transformed human retinal pigmented epithelial (RPE) cells were genetically engineered to express a fluorescent cell cycle reporter that enables accurate identification of each cell’s phase (i.e., G1, S, G2, or M) through time-lapse imaging (Stallaert et al., 2022a). Subsequently, the cells were fixed and subjected to iterative indirect immunofluorescence imaging (4i) to measure 48 key cell cycle effectors in 8,850 individual cells. A total of 246 single-cell features were derived from this imaging dataset, including protein expression and localization (e.g., nucleus, cytosol, perinuclear region, and plasma membrane), cell morphological attributes (such as nucleus and cell size and shape), and microenvironment characteristics (like local cell density), ultimately generating a comprehensive cell cycle signature for each cell within the population.

In their study, Stallaert et al. (2022a) narrowed the features to a set of 40 that most accurately predicted cell cycle phase (refer to Figure S1 panel A in Stallaert et al. (2022a)). Thus, the reduced dataset has a sample size of n=8,850 and an ambient dimension of d=40. Our goal is to decrease the dimension to $d^{\prime }=2$ for visualization purposes, while maintaining the four clusters that correspond to the four phases (G1, S, G2, M) and the cyclic structure: G1→S→G2→M→G1. To account for the diverse units of the 40 selected features, we applied z-score normalization to the data.

**Figure 5:**
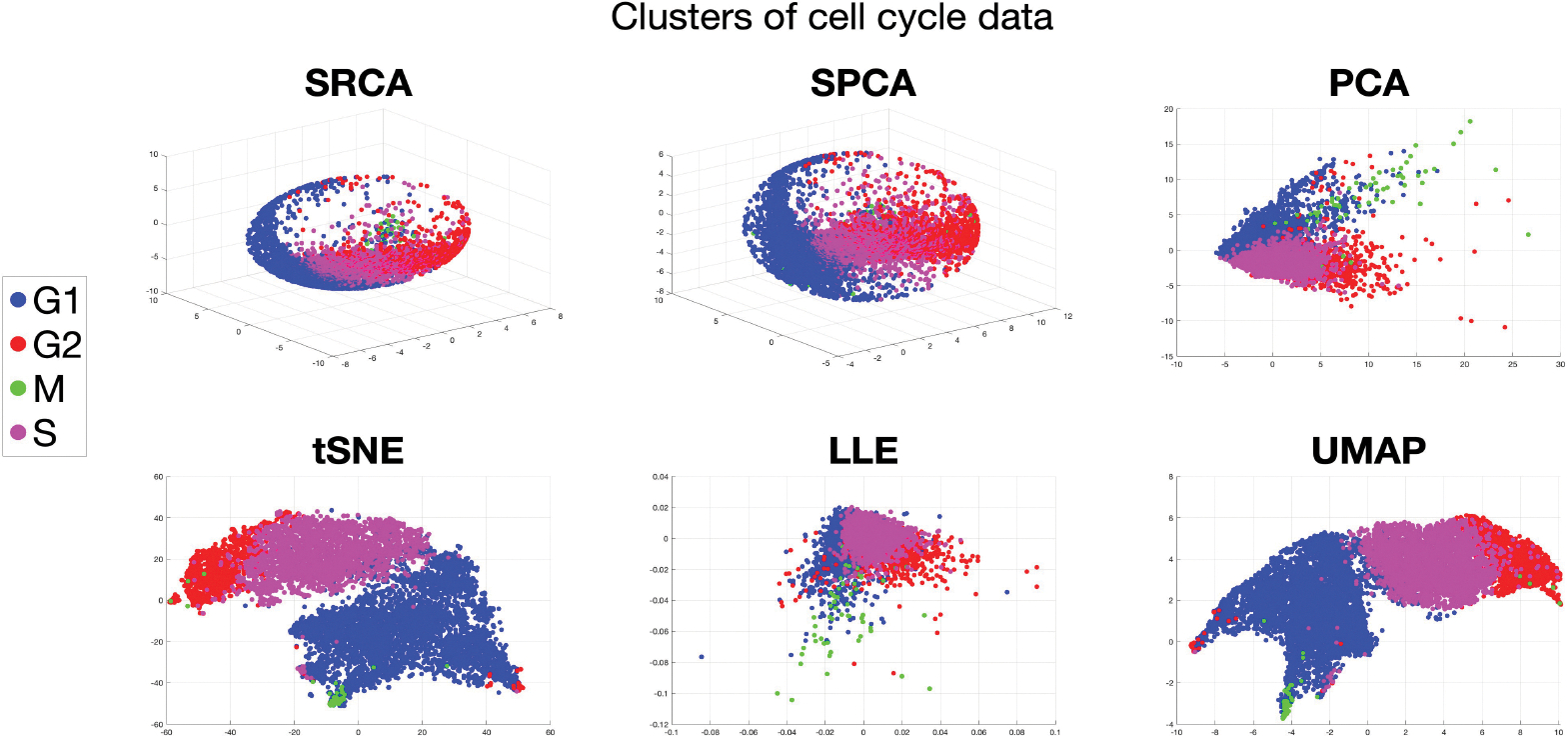
Cluster structures for human cell cycle, $d^{\prime }=2$, colored by phase.

Figure 5 displays the visualization of cell cycle data, with colors representing different cell cycle phases. While all algorithms can distinguish the four phases, PCA, tSNE, LLE, and UMAP fail to capture the cyclical structure. For example, the green points (M) should be located between the blue points (G1) and red points (G2); and magenta points (S) should be opposite to green points. In contrast, both SRCA and SPCA successfully recover the cyclical structure on 2-dimensional spheres. To compare SRCA and SPCA, we assess the MSE, as shown in the first column of Table 4.

Given that the true structure is cyclical, clustering metrics that depend on linear structures, such as the Silhouette score, are not suitable for this example. Instead, we use external biological information to validate our findings. Among the four phases, G1 cells are known to possess greater degrees of freedom (Chao et al., 2019; Stallaert et al., 2022b), leading us to anticipate that the variance of samples within G1 will be the largest among the four phases, with variances for G2 and M being similar and variance for S being the smallest. The variance is defined as the distance between each sample and the cluster mean, so a larger variance indicates a more dispersed distribution of points within that cluster. The final four columns in Table 4 demonstrate that SRCA more accurately captures the heterogeneity of cell activities across different phases.

In addition to the spherical structural preservation property of SRCA, another benefit of applying SRCA to cell cycle data is its interpretability. Previous attempts to model cell cycle data have relied on manifold estimation methods such as tSNE and UMAP (McInnes et al., 2018), where the low dimension representations are not biologically interpretable. In contrast, SRCA approximate the spherical structure within the original high dimensional space, so that the low dimension representations can be linked back to biolgically meaningful features. Moreover, given the widespread occurrence of cyclical data in biology (circadiam rhythms, hormonal oscillations, (Hogenesch and Ueda, 2011; Kubota et al., 2012)) , we believe that our method could have widespread application and impact when the underlying data are spherical in nature.

### 4.5 Parameter Selection

It is a separate but important problem to select (or tune) the parameter of both classical and modern DR methods. For classical DR methods, like PCA or MDS, the parameter usually has a explicit geometric interpretation. For modern non-linear DR methods, like tSNE and UMAP, the parameters affect both reproducibility and interpretability of the resulting dimension-reduced dataset.

The first parameter that dictates the behavior of most DR methods is the retained dimension $d^{\prime }$, which can be determined by subsequent purpose (e.g., the tSNE and UMAP usually take $d^{\prime }=2,\;3$ for visualizations).

The second parameter is the choice of rotation methods, which is highly data dependent and affects the clustering and visualization most. Regarding the MSE performance, Table 5 investigates the performance of SRCA with different kinds of rotations. We can see that the PCA rotation usually gives a reasonable result in terms of MSE performance. Both PCA and quartimax rotations used along with SRCA method outperforms dimension reduction of PCA and SPCA separately. We have similar observations for some other datasets with n>d (e.g., Leaf, PowerPlant, etc., see Supplement D).

The choice of rotation methods could also be made to accommodate the type of noise in observations. In the situation where the tail behavior of the noise is close to Gaussian and the W is known (or, by default I), PCA is our default choice; but in the situation where the noise is non-Gaussian and we do not have much knowledge for W, then ICA (Hyvärinen and Oja, 2000) is a better alternative.

Based on the empirical evidence obtained from real datasets (e.g., Table 5), we recommend using PCA rotation as a default, but other types of rotations can be useful for specific datasets, if desired (Jolliffe, 1995).

We summarize the observations from above experiments in sections 4.1 to 4.5. Although SRCA is the slowest in terms of computational time among SRCA, SPCA and PCA, it is rather fast compared to some non–linear methods like Isomap.

When the retained dimensions $d^{\prime }<min\{d,n\}$, SRCA behaves very similarly to SPCA in terms of the MSE, both outperform PCA alone across different real datasets. The interesting observation is that SRCA out-competes both of them in some simple but geometrically nontrivial examples like the ones in Supplement B, especially in clustering tasks. In most cases, PCA rotation is satisfactory, although considering other rotation methods may further improve the performance of SRCA.

When $d>d^{\prime }\geq n$, SRCA outperforms PCA, SPCA and other non-linear DR methods in terms of the coranking scores across different real datasets. Only SRCA yields consistently better dimension reduction results when $d^{\prime }<n$ and $d^{\prime }\geq n$.

## 5. Discussion

In this paper, we propose a novel DR method by proposing a rotation-based method with a geometric-induced loss function that minimizes the point-to-sphere distance from original to target spaces. Our motivation is to get the dimension reduction for spherical datasets (or datasets with spherical and elliptical structures) to respect the geometry in the original space. Its variant also works with a general weight matrix W and a sparsity penalty ξ. The proposed method is statistically principled, and is theoretically guaranteed to perform well asymptotically.

Unlike traditional DR methods like PCA and MDS, SRCA works smoothly with a stable performance even when $d\geq d^{\prime }+1>n$ which is extremely important in biomedical data DR, especially in gene expression data (e.g.,GTEx). Accompanying generalized algorithms for SRCA are also developed, with detailed convergence and a straightforward parallel potentiality for real-world practice. SRCA is related to PCA and SPCA but also generalizes the former into a spherical setting and the latter one into a one-step procedure. Most importantly, SRCA removes the $d^{\prime }<n$ requirements in these predecessors in a unified framework using novel loss functions.

Compared to non-linear methods, SRCA has geometrical interpretation and practical convenience. Its unique binary search also allows parallelizations when applied to big datasets. A comprehensive experimental study of SRCA against a collection of state-of-art DR methods has been done with detailed qualitative and quantitative measures, revealing the superiority of SRCA.

SRCA stands out for applying spherical dimension reduction to cell cycle data for the first time, revealing new biological insights by utilizing the data’s spherical characteristics. Considering the commonality of cyclical biological data (e.g., circadian rhythms, hormonal cycles), our method has the potential for broad use and significant impact in cases where the data is inherently spherical.

There are several directions of future work that we wish to pursue. For example, it is of great interest to see how geometric or topological loss function DR methods perform in data visualization (Sigmund, 2001; Nigmetov and Morozov, 2022). Another possible future work of estimating better rotation matrix as mentioned in Section 2.3 may inspire advanced optimization methods for non-convex problems. Novel regularization strategies may be necessary to maintain computational feasibility and ensure meaningful solutions. On the theoretical end, we would like to explore the convergence of our algorithm with the $l_{1}$ penalty, estimating the sparsity, and establish a non-asymptotic bound for our estimates. Our current technique focuses on but is not limited to spherical datasets. Similar designs of loss functions can be generalized to a wider variety of spaces like symmetric spaces (Li et al., 2020) using Lie group theory.

## Supplementary Material

1

## Figures and Tables

**Table 1:** Conceptual categorization of selected dimension reduction methods

	Local	Global
Manifold learning	LLE, tSNE, UMAP	MDS, Isomap, GPLVM
Manifold estimation	PCurv, GMRA, LPE, SAME, Spherelets	PCAs, SPCA, SRCA

**Table 2:** MSE for different experiments.

Dataset	Method/*d*′ =	1	2	3	4

Banknote	PCA	15.6261	6.3356	1.9479	

	SPCA	16.3717	8.1004	1.7348	
	
	SRCA	**13.439**	**5.5088**	**1.0743**	

Power Plant	PCA	222.2971	55.4460	23.5173	**2.9957**

	SPCA	162.8865	102.1006	45.5793	41.5251
	
	SRCA	**150.8041**	**52.1439**	**19.8839**	3.279

User Knowledge	PCA	0.1921	0.1253	0.0718	0.0311

	SPCA	0.1465	0.0893	0.0477	0.0148
	
	SRCA	**0.1458**	**0.0887**	**0.0471**	**0.0142**

Ecoli	PCA	0.076693	0.035222	0.020522	**0.00756**

	SPCA	**0.047776**	0.032948	0.019648	0.01136
	
	SRCA	0.076660	**0.032799**	**0.018332**	**0.00756**

Concrete	PCA	6.4469	4.4035	2.9539	1.7177

	SPCA	5.2285	3.4857	2.1825	0.9975
	
	SRCA	**5.2190**	**3.4745**	**2.1726**	**0.9862**

Leaf	PCA	8.2929	4.1102	2.0144	1.2810

	SPCA	5.2445	3.1907	2.4377	1.2608
	
	SRCA	**5.2223**	**3.1599**	**1.8433**	**1.1025**

Climate	PCA	1.4100	1.3204	1.2323	1.1450

	SPCA	1.3563	1.2648	1.1781	1.0907
	
	SRCA	**1.3554**	**1.2646**	**1.1780**	**1.0905**

**Table 3:** Clustering performance measures for Ecoli

Index	Baseline	SRCA	SPCA	PCA	LLE	tSNE	UMAP
SC	0.257	0.267	0.260	0.200	0.209	0.293	0.290
CHI	133	192	190	215	46.6	376	376
DBI	1.49	1.59	1.58	2.56	2.40	1.37	1.32

**Table 4:** Quantitative metrics of SRCA and SPCA for cell cycle data

	MSE	Var(G1)	Var(S)	Var(G2)	Var(M)
PCA	22.934	575	81	1297	3631
SPCA	22.252	566	110	276	763
SRCA	**22.098**	**633**	117	**434**	**475**

**Table 5:** MSE of Banknote (see Supplement D) for different rotation methods in the SRCA procedure. We also include two other DR methods PCA and SPCA to compare against SRCA. The first row records the DR methods (PCA, SPCA and SRCA); the second row records the optimal rotation method used by SRCA.

	PCA	SPCA	SRCA						
*d*′			PCA	varimax	orthomax	quartimax	equamax	parsimax	ICA
1	15.6	16.4	13.4	18.4	18.5	14.9	27.5	26.6	14.5
2	6.34	8.10	5.51	6.19	6.19	5.79	13.2	13.4	5.36
3	1.95	1.73	1.07	1.07	1.07	1.07	1.07	1.07	1.14
