# Supplementary material for: Spherical Rotation Dimension Reduction with Geometric Loss Functions
Source: J Mach Learn Res. Author manuscript; Available in PMC 2026 Mar 28. (PMC13028601)
Supplement: 1 [file NIHMS2118849-supplement-1.pdf]

## Appendix A. Orthogonal Loop Examples

Figure 6 shows orthogonal circles parallel to $xz$ and $xy$ planes respectively in $\mathbb{R}^3$. The projection of these two circles to the principal axes given by PCA is shown in Figure 7, where only one circular structure is retained in the reduced dataset while the other circular structure is completely destroyed

In Figure 8, we have the same but each coordinates is perturbed by a Gaussian noise with mean zero and different noise variances. As the noise variance increases, we observe that the topological structure of this example of two orthogonal loops becomes less and less obvious. We can see that SRCA is consistently achieving the lowest matched MSE defined in Section 4.1, while both SPCA and SRCA preserves the topological structure relatively well. It becomes evident that that SPCA and SRCA method better respect the topology of the original dataset under the same $d'$. PCA does not retain the circular structure, but SRCA puts both circles onto a larger 1-sphere congruent to $\mathbb{S}^1$.

In Table 6, we provide MSE for more settings of noise variances to show the MSE from each different DR methods. It can be observed that PCA becomes worse quickly in terms of MSE.

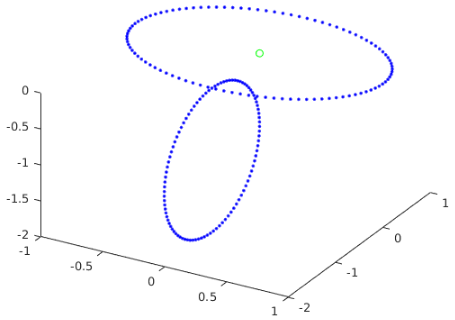


Figure 6: An example where PCA fails, adapted from Luo et al. (2021). Note that the two orthogonal circles only intersect at one point. The DR results are summarized in Figure 7

| Noise Var. | 0 | 0.01 | 0.05 | 0.10 | 0.20 | 0.40 | 1.00 |
|---|---|---|---|---|---|---|---|
| PCA | 0.24750 | 0.24889 | 0.25634 | 0.26990 | 0.31126 | 0.45045 | 1.2653 |
| SRCA | 0.10408 | 0.10421 | 0.10623 | 0.11237 | 0.13668 | 0.21834 | 0.64711 |
| SPCA | 0.12758 | 0.12764 | 0.12925 | 0.13448 | 0.15585 | 0.22861 | 0.65268 |

Table 6: MSE for different DR methods performed on the same orthogonal loop dataset but with different noise variances in the Gaussian perturbation.

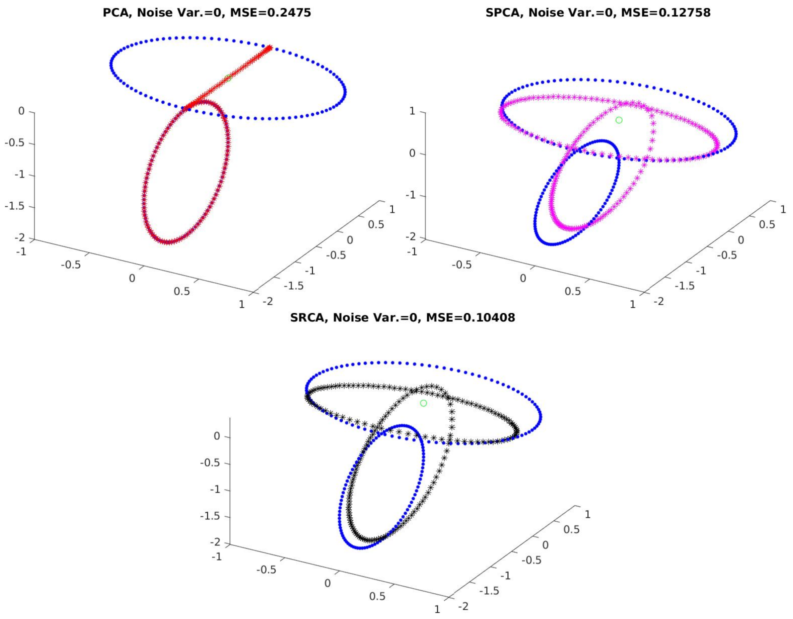


Figure 7: The dimension-reduced dataset from the example shown in Figure 6. The green dot represents the origin $(0, 0, 0)$. The original dataset is represented by blue points. On the left panel, the PCA dimension-reduced dataset is represented by red stars. On the middle and right panels the dimension-reduced dataset processed by SPCA and SRCA, is represented by magenta and black stars, respectively.

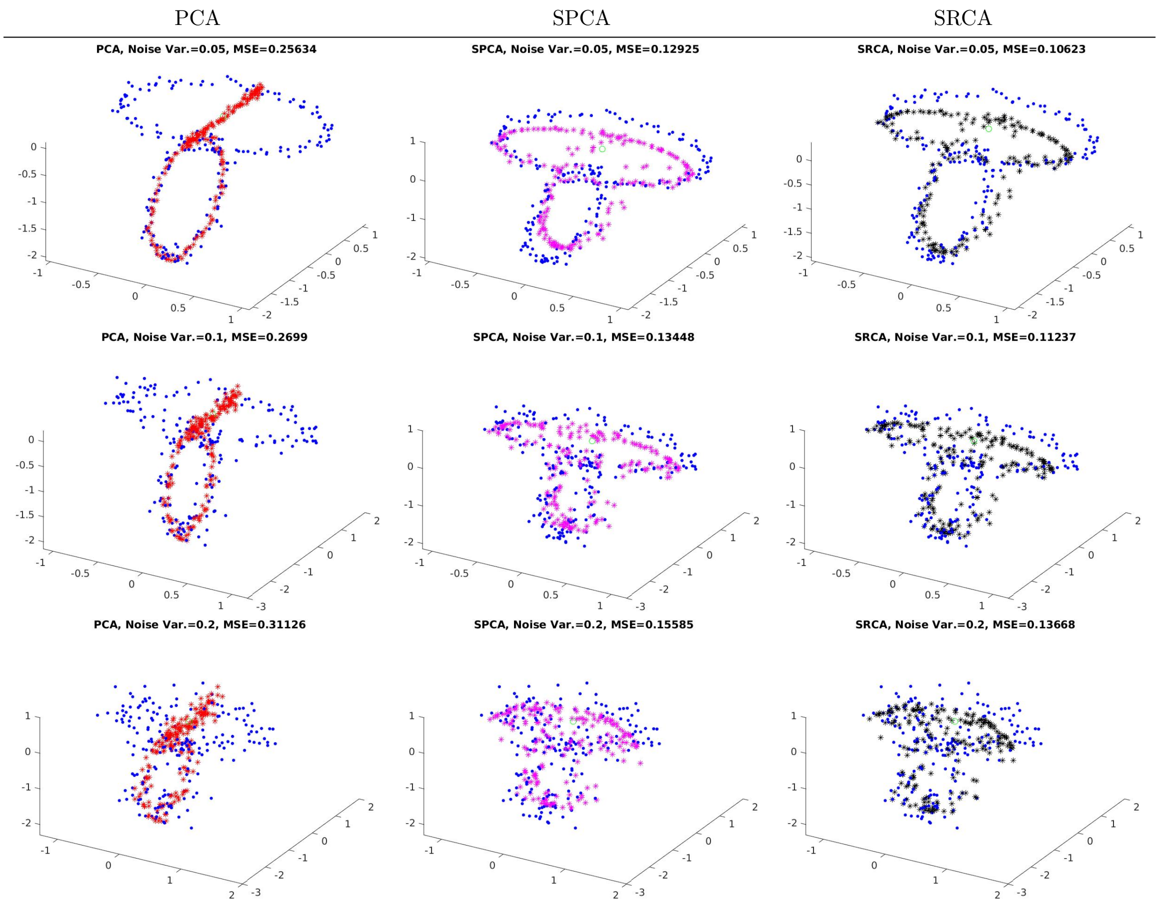


Figure 8: On the left columns we show the original dataset in blue points, and the PCA dimension-reduced dataset in red stars. On the middle column we show the original dataset in blue points, and the SPCA dimension-reduced dataset in magenta stars. On the right column we show the original dataset in blue points, and the SRCA dimension-reduced dataset in black stars.

## Appendix B. Other Synthetic Examples

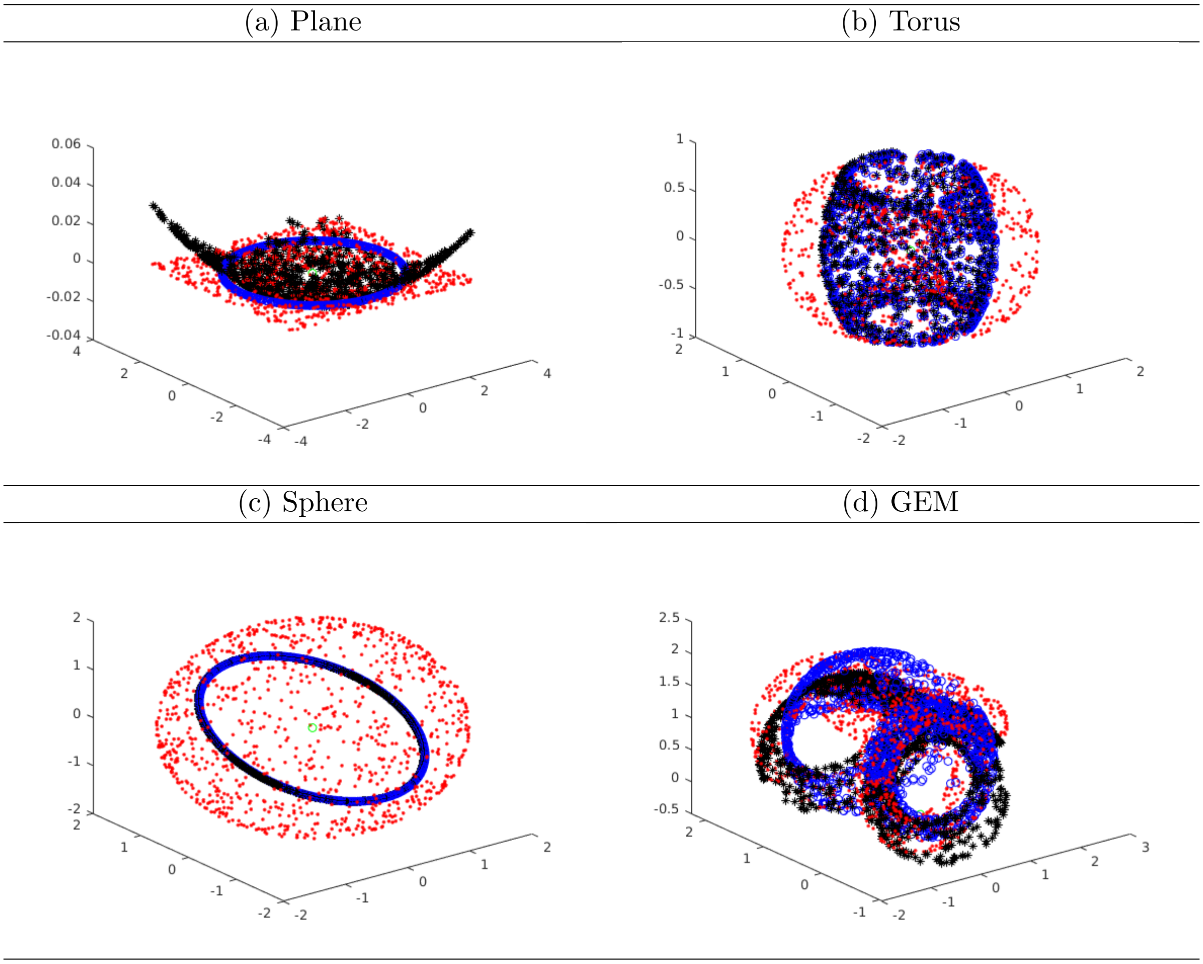


Figure 9: The datasets for the basic example. In these examples the datasets are observed in $\mathbb{R}^3$ ($d = 3$) and we want to reduce the dataset by one dimension ($d' = 2$). In each of the above panels, the red solid dots are the original datasets sampled from a uniform distribution lying on (a) plane (b) torus (c) sphere (d) triple torus without interior intersection (GEM). The blue circles are the points in reduced dataset obtained by SPCA. The black stars are the points in reduced dataset obtained by SRCA. We do not display the result of PCA in these figures.

In this appendix, we consider several basic examples we use to illustrate the difference between PCA, SRCA, and SPCA. Below, we provide the quantitative measures for each of these examples and the coranking summaries. In (a) plane, we uniformly sample points $(x_1, x_2, 0)$ from $[-3, 3] \times [-3, 3] \times \{0\}$. In (b) torus, we take the parameterization

$$((R_1 + R_2 \cos\theta)\cos\phi, (R_1 + R_2 \cos\theta)\sin\phi, R_2 \sin\theta)$$

where $R_1 = 1/2$ and $R_2 = 1/3$. The parameters $(\theta, \phi)$ are uniformly sampled from $[0, 2\pi) \times [0, 2\pi)$. In (c) sphere, we take the canonical parameterization and uniform sampling on the

parameter space $[0, 2\pi) \times [0, 2\pi)$, which is equivalent to von-Mises Fisher distribution with concentration $\kappa = 0$. In (d) triple torus, we independently sampled 3 batches of points with equal sample sizes. Then we multiply each of these 3 batches with the following rotation matrices and add a translation vector:

$$R_1 = \begin{pmatrix} 1 & 0 & 0 \\ 0 & \cos\frac{\pi}{2} & -\sin\frac{\pi}{2} \\ 0 & \sin\frac{\pi}{2} & \cos\frac{\pi}{2} \end{pmatrix}, \tau_1 = \begin{pmatrix} 0 \\ 0 \\ 3 \end{pmatrix}.$$

$$R_2 = \begin{pmatrix} \cos\frac{\pi}{4} & 0 & \sin\frac{\pi}{4} \\ 0 & 1 & 0 \\ -\sin\frac{\pi}{4} & 0 & \cos\frac{\pi}{4} \end{pmatrix}, \tau_2 = \begin{pmatrix} 0 \\ 3 \\ 3 \end{pmatrix}.$$

$$R_3 = \begin{pmatrix} \cos 0 & -\sin 0 & 0 \\ \sin 0 & \cos 0 & 0 \\ 0 & 0 & 1 \end{pmatrix}, \tau_3 = \begin{pmatrix} 3 \\ 3 \\ 3 \end{pmatrix}.$$

The resulting dataset contains three tori, and it is not difficult to verify that these three tori do not have interior intersections. We scale the dataset by subtracting $(1, 1, 1)^T$ from each point and multiply 1/2 entry-wisely.

| | SRCA | SPCA | PCA | LLE | tSNE | UMAP |
|---|---|---|---|---|---|---|
| CC | 0.9999998 | 0.9998008 | 1.0000000 | 0.9983610 | 0.9774094 | 0.9906862 |
| AUC | 0.9995782 | 0.9898617 | 0.9998370 | 0.9604653 | 0.8670743 | 0.9094597 |
| WAUC | 0.9990011 | 0.9913487 | 0.9991016 | 0.9725947 | 0.8460439 | 0.8320849 |

Table 7: Performance scores and the coranking of plane.

| | SRCA | SPCA | PCA | LLE | tSNE | UMAP |
|---|---|---|---|---|---|---|
| CC | 0.8222110 | 0.8208433 | 0.9777033 | 0.9751233 | 0.8523848 | 0.9072153 |
| AUC | 0.6217176 | 0.6194763 | 0.8317488 | 0.8251250 | 0.6344541 | 0.7257927 |
| WAUC | 0.6025524 | 0.6033849 | 0.6104916 | 0.6092178 | 0.7430941 | 0.6707993 |

Table 8: Performance scores and the coranking of torus.

| | SRCA | SPCA | PCA | LLE | tSNE | UMAP |
|---|---|---|---|---|---|---|
| CC | 1.0000000 | 1.0000000 | 0.8026803 | 0.7262926 | 0.6762080 | 0.6810310 |
| AUC | 0.9999992 | 1.0000000 | 0.5585561 | 0.4876606 | 0.5171233 | 0.5696564 |
| WAUC | 0.9999998 | 1.0000000 | 0.4953096 | 0.4792300 | 0.7312668 | 0.7270227 |

Table 9: Performance scores and the coranking of sphere.

| | SRCA | SPCA | PCA | LLE | tSNE | UMAP |
|---|---|---|---|---|---|---|
| CC | 0.9986899 | 0.7407685 | 0.9987456 | 0.9963411 | 0.5697951 | 0.9438497 |
| AUC | 0.9509051 | 0.5618742 | 0.9518870 | 0.9205247 | 0.4646542 | 0.8042776 |
| WAUC | 0.7067388 | 0.5204686 | 0.7057702 | 0.6973970 | 0.7077429 | 0.6735852 |

Table 10: Performance scores and the coranking of GEM.

From the performance evaluation table above, we can see that:

1. In the plane example, all linear and non-linear DR methods performs well in terms of performance evaluation measures and coranking summaries.
2. In the torus example, neither SPCA nor SRCA perform as good as other DR methods in terms of scores. However, in the visualization of the reduced dataset, we can see that both SPCA and SRCA preserve the structure of torus pretty well.
3. In the sphere example, both SPCA and SRCA give almost identical results, outperforming both the simplest PCA and the more sophisticated non-linear DR methods like tSNE and UMAP. In the visualization of the reduced dataset, we can see that both SPCA and SRCA reduce the data points on the sphere ($d = 3$) to points on a geodesic circle ($d' = 2$). This preserves the spherical nature of the dataset and yield a better result.
4. In the GEM example, the SRCA is the best in terms of almost all performance scores. A visual verification also reveals that the resulting reduced dataset preserves all three holes in the tori.

## Appendix C. SRCA Algorithms

In this section, we present the algorithm that solves our optimization problem (2) and (3). Algorithm 1 delineates the binary search strategy which exhausts $2^d$ possible subsets of $\{1, \cdots, d\}$ to find an optimal subspace. Algorithm 2 delineates the $l_1$-relaxation strategy which formulates the original problem (2) into an optimization problem (3) to find an optimal subspace.

Detailed explanation of algorithms in this section is as follows.

1. (Step 1: Get the empirical mean for $\mathcal{X}$.) Estimate the empirical mean $\bar{\mathcal{X}}$ for the dataset $\mathcal{X}$ in $\mathbb{R}^d$, and subtract the mean $\bar{\mathcal{X}}$ to make sure that the assumption of PCA is satisfied.

$$z_i = x_i - \frac{1}{n}\sum_{i=1}^{n} x_i = x_i - \bar{x}$$

2. (Step 2: Conduct the rotation.) We choose a rotation method to construct rotation matrix $R$ based on the datset $\mathcal{X}$. Then we rotate the dataset $\mathcal{X}$ to standard position $(\mathcal{X} - \bar{\mathcal{X}})R$. Here we use a chosen rotation matrix $R$ (PCA, ICA or other kinds of optimal rotation) to rotate the sphere so that all its axes are parallel to the coordinate

axes.
For PCA rotation, let the covariance matrix $\text{cov}(z_i) = R\Lambda R^T$ where $\Lambda$ is diagonal and the rotation matrix $R$ is orthogonal.

$$y_i = Rz_i$$

3. (Step 3: Binary search for the best $d' + 1$ axes.) In this step we perform dimension reduction. Now that we can assume that the axes of the sphere (or ellipsoid) are parallel to the coordinate axes. We solve the binary optimization problem (2) with $W = I$ (or other $W$ if extremely skewed dataset is observed) to choose the optimal directions to retain. In this step we would find the optimal $v_{\text{opt}}$ and hence the optimal index set $\mathcal{I}_{\text{opt}}$.
   In this optimization problem, as we stated in (2) above, we conduct dimension reduction by minimizing the loss function based on the point-to-ellipsoid distance to the estimated sphere $S_{\mathcal{I}}$.

   The optimization problem is spelled out as (4).

4. (Step 4: Eliminate the un-chosen dimensions.) This simply sets drop the un-selected dimension not in $\mathcal{I}_{\text{opt}}$, equivalently, we find the $v_{\text{opt}}$ in the notation of problems (2) and (3).

   (a) In the standard form (2), we may let $\eta = v_{\text{opt}}$ be the binary vector such that $\|v_{\text{opt}}\|_{l_0} = d' + 1$.

   (b) In the $l_1$-relaxed form (3), the $v_{\text{opt}}$ would have $l_1$-norm less or equal than $d' + 1$ but not binary entries. We construct a binary vector $\eta$ such that only the first leading $d' + 1$ entries with the largest absolute values in $v_{\text{opt}}$ are 1; and the rest entries are 0.

5. (Step 5: Re-estimate the center and radius.) Only in the problem (3), we re-estimate the center $c_{\text{opt}}$ and radius $r_{\text{opt}}$ using the same loss function but a fixed $v_{\text{opt}}$. In standard problem (2), we use the center $c_{\text{opt}}$ and radius $r_{\text{opt}}$ from step 3.

6. (Step 6: Project and rotate the sphere back into full space.) After we choose the dimension and axes, we project the dataset onto a sphere with the center $c_{\text{opt}}$ and radius $r_{\text{opt}}$ and add back the empirical mean $\bar{\mathcal{X}}$. More specifically, we project the datapoints $x_i$ to the sphere $S(c, r)$ as in (5) and then we simply we rotate the resulting dimension-reduced dataset back, using the inverse of the same rotation matrix $R$.

**Algorithm 2:** SRCA dimension reduction algorithm with $l_1$ relaxation

**Data:** $X$ (data matrix consisting of $n$ samples in $\mathbb{R}^d$)
**Input:** $d'$ (the dimension of the sphere), $W$ (the covariance weight matrix, by default $W = I_d$), $\xi$ (optional, the sparse penalty parameter), rotationMethod (the method we use to construct the rotation matrix).
**Result:** $\hat{c}$ (The estimated center of $S_{\mathcal{I}}$ in $\mathbb{R}^d$), $\hat{r}$ (The estimated radius of $S_{\mathcal{I}}$), $\mathcal{I}_{opt}$ (The optimal index subset)
`GetRotation` ($X$,rotationMethod) and `ProjectToSphere` ($X$,$c$,$r$,$k$) are identically defined in Algorithm 1.
**begin**
  Standardize the dataset by subtracting its empirical mean $X = X - \bar{X}$
  Construct a rotate matrix $R =$ `GetRotation` ($X$,rotationMethod)
  $X_{rotated} = X * R$
  Solve the optimization problem (3) with respect to $c, r$ and $v$
  Denote the solution as $c_0, r_0, v_{opt}$
  Construct $\mathcal{I}$ that contains the largest/leading $d' + 1$ coordinates of $v_{opt}$.
  Solve the optimization problem (2) with respect to $c, r$ with a fixed $\mathcal{I}$
  Denote the solution as $c_{opt}, r_{opt}$
  $\hat{c} = c_{opt} \cdot \eta * R^{-1} + \bar{X}$
  $\hat{r} = r_{opt}$
  $X_{rotated}(:, \mathcal{I}) \leftarrow 0$
  $X_{rotated} \leftarrow$ `ProjectToSphere` ($X$,$\hat{c}$,$\hat{r}$,$k$)
  $X_{output} \leftarrow X_{rotated} * R^{-1} + \bar{X}$
**end**

## Appendix D. Dataset Selection

We select datasets for high- and low-dimensional scenarios, covering both $n \geq d$ and $n < d$ as detailed below. Thanks to the binary search scheme we designed in our SRCA algorithm, SRCA can be parallelized when an even larger $n$ (e.g., $n = 100,000$) presents. Neither PCA nor non-linear methods we consider below can be generalized to a quite large $n$ in an obvious way.

The tSNE (Van der Maaten and Hinton, 2008) has known problems of not distance-preserving and creating false clusters in the dimension-reduced dataset (Schubert and Gertz, 2017). The UMAP (McInnes et al., 2018) has known problems of being sensitive to outliers and require the user to have a good understanding of their distance metrics to interpret the dimension-reduced dataset. To highlight the advantage of our method with a geometric loss function, we also choose labeled datasets to study the structure-preserving properties and datasets that require careful normalization.

We use several public datasets for our numerical experiments, but consider only datasets with continuous variables as its features, as we recognized that dimension reduction for datasets with discrete (categorical and integer) or mixed type variables as their attributes is a different problem (Schölkopf et al., 1997, 1998).

Before the analysis of our experiments, we shall briefly introduce our datasets:

- Source

  - UCI repository (`https://archive.ics.uci.edu/ml`): Banknote, Climate, Concrete, Ecoli[1], Leaf, PowerPlant, UserKnowledge.
  - Microarray: Alon (Alon et al., 1999).
  - GTEx (`https://gtexportal.org/home/`).

- Sample size
  We understand that a valid dimension reduction method should have reasonable performance regardless of the size of the underlying datasets. We choose a wide range of datasets with sample sizes varying from 100 to 10,000. In the table below, we show the code for each dataset with its sample size and dimension ($n \times d$).

| $n > d$ | $n \leq d$ |
|---|---|
| Banknote ($1372 \times 4$), UserKnowledge ($403 \times 5$), | Kidney Medulla ($4 \times 500$), |
| Ecoli ($336 \times 7$), Concrete ($1030 \times 8$), | Fallopian Tube ($9 \times 500$), |
| Climate ($540 \times 18$), Leaf ($340 \times 14$), | Cervix Endocervix ($10 \times 500$),. |
| PowerPlant ($9568 \times 5$), | Alon ($62 \times 2000$), . |

- Dimensionality
  It is of central importance to recognize that the relation between the sample size $n$ and the original dimension $d$ would affect dimension reduction methods. In fact, the sparse PCA (Erichson et al., 2020) were developed to take the sparsity (i.e., $n < d$) of the dataset into consideration. SRCA has a natural sparsity penalty parameter in the loss function $\mathscr{L}$ we designed. To see the performance of different methods on dense ($n > d$) and sparse ($n \leq d$) data, we also include both kinds of datasets in the above selection.

- Normalization
  When the attributes or features of the dataset have high mutual correlation or relationships with one another (e.g., total expenditure cannot exceed total income of an individual), normalization would introduce problems like distortion of correlation and violation of relationships. When the attributes or features of the dataset are uncorrelated or independent, normalization would convert all features to (relatively) the same scale. We include datasets that require normalization and those that do not require normalization.
  - Not normalized: Banknote, Ecoli, PowerPlant, UserKnowledge, Climate.
  - Normalized: Alon, Concrete, Leaf, GTEx.

Finally, we shall point out that we have not covered any dataset with a high $d$ and large $n$. Our exploratory experiments found that the performance of existing dimension methods, including modern methods, has suffered from a very high computational cost. It is a separate problem to study how to perform dimension reduction on a large high-dimensional dataset.

1. Three groups among them with sample size smaller than 5 are removed for visual convenience. The five groups left are cytoplasmic proteins (cp), inner membrane proteins without a signal sequence (im), inner brane proteins with an uncleavable signal sequence (imU), other outer membrane proteins (om) and periplasmic proteins (pp).

Decomposition-based methods like PCA, multi-dimensional scaling (MDS) and truncated singular value decomposition (SVD) become exceedingly slow for a dataset with large $n$ and $d$. Manifold learning based methods like tSNE, UMAP and IsoMap (Tenenbaum et al., 2000) have some variation in computational time due to their stochastic nature, but they are all rather slow. Therefore, we would leave this type of dataset as a separate problem that we do not experiment in the current paper.

## Appendix E. Coranking Performance Comparison for Section 4.3

| | SRCA | SPCA | PCA | LLE | tSNE | UMAP |
|---|---|---|---|---|---|---|
| CC | 0.987 | 0.925 | 0.988 | 0.833 | 0.640 | 0.635 |
| AUC | 0.869 | 0.774 | 0.860 | 0.598 | 0.469 | 0.459 |
| WAUC | 0.644 | 0.582 | 0.626 | 0.503 | 0.694 | 0.600 |

Table 11: Coranking performance scores of Banknote

| | SRCA | SPCA | PCA | LLE | tSNE | UMAP |
|---|---|---|---|---|---|---|
| CC | 0.270 | 0.270 | 0.262 | 0.464 | 0.215 | 0.124 |
| AUC | 0.152 | 0.152 | 0.148 | 0.225 | 0.147 | 0.102 |
| WAUC | 0.134 | 0.132 | 0.0864 | 0.105 | 0.141 | 0.118 |

Table 12: Coranking performance scores of Ecoli

| | SRCA | SPCA | PCA | LLE | tSNE | UMAP |
|---|---|---|---|---|---|---|
| CC | 0.987 | 0.815 | 0.987 | 0.928 | 0.620 | 0.847 |
| AUC | 0.886 | 0.605 | 0.886 | 0.731 | 0.446 | 0.651 |
| WAUC | 0.485 | 0.416 | 0.485 | 0.447 | 0.611 | 0.528 |

Table 13: Coranking performance scores of PowerPlant

| | SRCA | SPCA | PCA | LLE | tSNE | UMAP |
|---|---|---|---|---|---|---|
| CC | 0.219 | 0.211 | 0.219 | 0.183 | 0.134 | 0.180 |
| AUC | 0.0780 | 0.0853 | 0.0785 | 0.0700 | 0.0707 | 0.0635 |
| WAUC | 0.0935 | 0.0952 | 0.0948 | 0.0762 | 0.106 | 0.100 |

Table 14: Coranking performance scores of Leaf

| | SRCA | SPCA | PCA | LLE | tSNE | UMAP |
|---|---|---|---|---|---|---|
| CC | 0.730 | 0.798 | 0.693 | 0.585 | 0.483 | 0.592 |
| AUC | 0.416 | 0.456 | 0.379 | 0.338 | 0.231 | 0.384 |
| WAUC | 0.256 | 0.3233 | 0.251 | 0.212 | 0.214 | 0.328 |

Table 15: Coranking performance scores of Alon.

## Appendix F. Sparse Penalty

| $\xi$ | $10^{-1}$ | $10^{-2}$ | $10^{-3}$ | $10^{-4}$ | $10^{-5}$ | 0 |
|---|---|---|---|---|---|---|
| CC | 0.704 | 0.727 | 0.730 | 0.730 | 0.730 | 0.730 |
| AUC | 0.408 | 0.416 | 0.416 | 0.416 | 0.416 | 0.416 |
| WAUC | 0.261 | 0.256 | 0.256 | 0.256 | 0.256 | 0.256 |

Table 16: Coranking performance scores for different $\xi$'s on the Alon dataset, $d' = 2$.

Here, we provide a new version of SRCA with sparse penalty, which only involves an additional penalty term in the loss function we designed. Recall that the objective loss function with a weighted matrix $W$ in our method is

$$d(x_i, S_{\mathcal{I}}(c,r))^2 = (x_i - c)^T W (x_i - c) + r^2 - 2r\sqrt{(x_i - c)^T \sqrt{W}^T I_{\mathcal{I}} \sqrt{W}\,(x_i - c)}$$

which involves only the point-to-sphere distance from $x_i$ to the estimated sphere surface $S_{\mathcal{I}}$ (based on dataset $\mathcal{X} = \{x_1, x_2, \cdots, x_n\}$). One problem we wish to address when there exists sparsity in the dataset in the procedure of dimension reduction, is that we want the sparsity being preserved.

To be more precise, if $x_i$'s have most coordinates zeros except for a few, then we want the reduced dataset $\hat{x}_i$ to have a similar property. This can be achieved by penalizing $\|I_{\mathcal{I}}(x_i - c)\|_1$ in the optimization problem, which encourages the estimated sphere so that the data are in the affine subspace centered at $c$ while parallel to the coordinate planes. This is different from the $l_1$ relaxation we propose above. The $l_1$ relaxation we proposed above is an approximation to the constraints we imposed on the binary optimization problem. Here, we directly penalize the reduced coordinates. The corresponding optimization problem is:

$$\begin{aligned}\min_{c\in\mathbb{R}^d, r\in\mathbb{R}^+} &\sum_{i=1}^{n} \Big((x_i - c)^T W (x_i - c) + r^2 \\ &-2r\sqrt{(x_i - c)^T \sqrt{W}^T v^T I v \sqrt{W}\,(x_i - c)}\Big) + \xi \|I_{\mathcal{I}}(x_i - c)\|_{l_1}, \quad (10)\\ &\text{s.t. } \|v\|_{l_1} \le d' + 1, \xi > 0, \quad (11)\end{aligned}$$

with a tuning parameter $\xi > 0$. $\|v\|_{l_1} \le d' + 1$ can be $\|v\|_{l_1} = d' + 1$. This feature of $l_1$ constraint allows us to perform dimension reduction in a high-dimensional input space with SRCA. We want to consider the penalty parameter $\xi$ that controls the retained dimension $d'$ when $l_1$ approximation is in place as defined in (10). When a strict binary search like

$l_0$ optimization in (4) is used, the penalty is usually not needed. However, the caution we shall take here is that the selection of sparsity penalty parameter $\xi$ should roughly be at the same magnitude as the loss function in order to function properly.

The tuning parameter $\xi > 0$ is part of the objective function, instead of the constraints. In some applications, the penalty term can also be replaced with $\xi \sum_{i=1}^{n} \|I_{\mathcal{I}} x_i\|_1$. In experiments, we found that SRCA is not sensitive to $\xi$. In very high-dimensional datasets (e.g., Alon (see Supplement D), GTEx), the choice of this parameter also affects the convergence speed in the execution of the optimization algorithm, a larger penalty parameter forces the numerical algorithm to converge slightly faster. Alternatively, we can also treat the choice of this parameter as a apriori tuning parameter of the loss function, whose values can be selected for different datasets using cross-validation.

## Appendix G. Spherical Estimation

The essence of SPCA (Li et al., 2022) can be summarized as a two-step procedure:

1. First, we utilize the principal component analysis (PCA) to find a subspace $V \subset \mathbb{R}^{d'+1}$ of retained dimension based on $\mathcal{X}$ and project $\mathcal{X}$ to $\hat{\mathcal{X}}$ in $V$.

2. Second, we perform a circular (or $d'$-dimensional spherical) regression[2] with the projected image $\hat{\mathcal{X}}$ onto $V$.

By selecting the principal components given by PCA, we find a subspace $V$ and determine the dimension of the $S$. By fitting a circular regression on a $d'$-dimensional sphere with the projected dataset $\hat{\mathcal{X}}$, we determine the center $c$ and radius $r$ of the spherical support.

Suppose the assumed sphere $S_V(c, r)$ is $d^2(x, c) = r^2$, whose dimensionality is determined by the PCA estimated linear subspace $V$. Two typical loss functions for the estimation of $c, r$ are:

$$\mathscr{L}(V, c, r) = \sum_{i=1}^{n} d^2(x_i, S_V(c, r))$$

where $d^2$ can be chosen as geometric or algebraic loss

$$\text{geometric loss} = \sum_{i=1}^{n} \left( \sqrt{(x_i - c)^T (x_i - c)} - r \right)^2$$

$$\text{algebraic loss} = \sum_{i=1}^{n} \left( (x_i - c)^T (x_i - c) - r^2 \right)^2$$

Following Li et al. (2022), we first assume that $V$ is determined (through PCA) and attempt to estimate the center $c$ and radius $r$ via a two-step gradient descent with both geometric and algebraic loss functions. Through the procedure of taking derivation, we observe and explain why an analytic solution for $c$ and $r$ is impossible in this SPCA setup in the end and how SRCA handles this problem.

2. Unfortunately, although methods in circular regression could be extended to spheres of intrinsic dimensions greater than 1, the term "circular regression" instead of "spherical regression" is adopted.

### G.1 Geometric Loss

Let us calculate the geometric loss first, the algebraic loss is calculated at the end. This function is a quadratic polynomial of radius parameter $r > 0$, $\mathscr{L}$ has a unique global (conditional) minimum in $r$ if $\hat{r} > 0$. When the $c$ is assumed fixed.

We calculate its gradient

$$\begin{aligned}\frac{\partial \mathscr{L}(c,r)}{\partial r} &= \sum_{i=1}^{n} \frac{\partial}{\partial r} d^2(x_i, S(c,r)) \\ &= \sum_{i=1}^{n} \frac{\partial}{\partial r} \left( \sqrt{(x_i - c)^T (x_i - c)} - r \right)^2 \\ &= \sum_{i=1}^{n} -2 \left( \sqrt{(x_i - c)^T (x_i - c)} - r \right)\end{aligned}$$

Setting this equation to zero, we have

$$\hat{r} = \frac{1}{n} \sum_{j=1}^{n} \sqrt{(x_j - c)^T (x_j - c)} \geq 0.$$

Plug this back into the $\mathscr{L}(c,r)$ we have

$$\begin{aligned}\mathscr{L}(c,\hat{r}) &= \sum_{i=1}^{n} d^2(x_i, S(c,\hat{r})) \\ &= \sum_{i=1}^{n} \left( \sqrt{(x_i - c)^T (x_i - c)} - \hat{r} \right)^2 \\ &= \sum_{i=1}^{n} \left( \sqrt{(x_i - c)^T (x_i - c)} - \frac{1}{n} \sum_{j=1}^{n} \sqrt{(x_j - c)^T (x_j - c)} \right)^2\end{aligned}$$

Although this cannot be simplified further (due to the fact that it is fourth power in $c$), we can still attempt to take its gradient

$$\begin{aligned}\frac{\partial \mathscr{L}(c,\hat{r})}{\partial c} &= \sum_{i=1}^{n} \frac{\partial}{\partial c} \left( \sqrt{(x_i - c)^T (x_i - c)} - \frac{1}{n} \sum_{j=1}^{n} \sqrt{(x_j - c)^T (x_j - c)} \right)^2, \\ &\text{where } \hat{r} = \frac{1}{n} \sum_{j=1}^{n} \sqrt{(x_j - c)^T (x_j - c)} \\ &= \sum_{i=1}^{n} 2 \left( \sqrt{(x_i - c)^T (x_i - c)} - \hat{r} \right) \cdot \\ &\left( \frac{\partial}{\partial c} \sqrt{(x_i - c)^T (x_i - c)} - \frac{1}{n} \sum_{j=1}^{n} \frac{\partial}{\partial c} \sqrt{(x_j - c)^T (x_j - c)} \right)\end{aligned}$$

The equation $\frac{\partial \mathscr{L}(c,\hat{r})}{\partial c} = 0$ would not have an analytic solution in general. However, with an appropriate gradient-based optimization method, for example, Gauss-Newton method with Levenberg-Marquardt correction (Chernov, 2010), the sequence of estimates of $c, r$ can be proven to converge to global minimum under the regularity condition. It is also not hard to observe why the insertion of $W$ into the $\sqrt{(x_i - c)^T W (x_i - c)}$ makes the gradient calculation even more intractable for the geometric loss function.

### G.2 Algebraic Loss

However, analytic solutions for a sphere estimation can be derived for algebraic loss. It can also generalize to ellipsoid (i.e., an algebraic loss can be solved analytically for the ellipsoid $x^T W x = r$)

$$\begin{aligned}
\frac{\partial \mathscr{L}(c,r)}{\partial r} &= \sum_{i=1}^{n} \frac{\partial}{\partial r} d^2(x_i, S(c,r)), \text{ algebraically} \\
&= \sum_{i=1}^{n} \frac{\partial}{\partial r} \left( (x_i - c)^T (x_i - c) - r^2 \right)^2 \\
&= \sum_{i=1}^{n} 2 \left( (x_i - c)^T (x_i - c) - r^2 \right) \cdot (-2r)
\end{aligned}$$

which is a cubic polynomial. $\frac{\partial \mathscr{L}(c,r)}{\partial r} = 0$ is analytically solvable in $r$, via Cardano-Viete's formula:

$$\begin{aligned}
0 &= \sum_{i=1}^{n} -2 \left( (x_i - c)^T (x_i - c) - r^2 \right) \cdot 2r \\
0 &= \sum_{i=1}^{n} \left( \left( x_i^T x_i - 2c^T x_i + c^T c \right) - r^2 \right) \cdot r \\
0 &= \sum_{i=1}^{n} \left( \left( x_i^T x_i - 2c^T x_i + c^T c \right) r - r^3 \right) \\
0 &= -n \cdot r^3 + \left[ \sum_{i=1}^{n} \left( x_i^T x_i - 2c^T x_i + c^T c \right) \right] \cdot r.
\end{aligned}$$

Write it into $x^3 + px + q = 0$ form:

$$\begin{aligned}
r^3 + \left[ -\frac{1}{n} \sum_{i=1}^{n} \left( x_i^T x_i - 2c^T x_i + c^T c \right) \right] \cdot r + 0 = 0 \\
p = -\frac{1}{n} \sum_{i=1}^{n} \left( x_i^T x_i - 2c^T x_i + c^T c \right), q = 0
\end{aligned}$$

The determinant $4p^3 + 27q^2 < 0$ obviously, the solution is

$$\begin{aligned}\hat{r}_k &= 2\sqrt{-\frac{p}{3}} \cdot \cos\left[\frac{1}{3}\arccos\left(\frac{3q}{2p}\sqrt{\frac{-3}{p}}\right) - \frac{2\pi k}{3}\right] \text{ for } k = 0, 1, 2. \\ &= 2\sqrt{\frac{1}{3n}\sum_{i=1}^{n}\left(x_i^T x_i - 2c^T x_i + c^T c\right)} \cdot \cos\left[\frac{1}{3}\cdot\frac{\pi}{2} - \frac{2\pi k}{3}\right]\end{aligned}$$

For the gradient with respect to the center $c$,

$$\begin{aligned}\frac{\partial \mathscr{L}(c,\hat{r})}{\partial c} &= \sum_{i=1}^{n} \frac{\partial}{\partial c} d^2(x_i, S(c,\hat{r})), \text{ algebraically} \\ &= \sum_{i=1}^{n} \frac{\partial}{\partial c}\left((x_i - c)^T (x_i - c) - \hat{r}^2\right)^2 \\ &= \sum_{i=1}^{n} 2\left(\frac{\partial}{\partial c}\left[(x_i - c)^T (x_i - c) - \frac{1}{n}\sum_{j=1}^{n}(x_j - c)^T (x_j - c)\right]\right) \\ &= \sum_{i=1}^{n} 2\left(\frac{\partial}{\partial c}\left[\left(x_i^T x_i - 2c^T x_i + c^T c\right) - \frac{1}{n}\sum_{j=1}^{n}\left(x_j^T x_j - 2c^T x_j + c^T c\right)\right]\right),\end{aligned}$$

and the equation $\frac{\partial \mathscr{L}(c,\hat{r})}{\partial c} = 0$ solves

$$\hat{c} = \frac{1}{2}\left(\sum_{i=1}^{n}(x_i - \frac{1}{n}\sum_{j=1}^{n} x_j)^T (x_i - \frac{1}{n}\sum_{j=1}^{n} x_j)\right)^{-1} \sum_{i=1}^{n}\left(x_i^T x_i - \frac{1}{n}\sum_{j=1}^{n} x_j^T x_j\right)(x_i - \frac{1}{n}\sum_{j=1}^{n} x_j).$$

Therefore, an algebraic loss would provide us a closed form solution to the estimate of both center $c$ and radius $r$.

### G.3 SPCA and SRCA Solution

Following the thought of the simultaneous estimation of $c, r$ and the dimension of the sphere (or equivalently, the linear subspace $\mathbf{V} \in \mathbb{R}^{d \times (d'+1)}$ where $S$ lives in), we can instead consider

the following geometric loss in one step

$$\begin{aligned}
\mathscr{L}(V,c,r) &= \sum_{i=1}^{n} d^2(x_i, S_V(c,r)) \\
&= \sum_{i=1}^{n} d^2(x_i, c+V) + \sum_{i=1}^{n} d^2(Pr_V(x_i), S_V(c,r)) \\
&= \sum_{i=1}^{n} \|x_i - c - VV^T(x_i - c)\|^2 + \sum_{i=1}^{n}(\|Pr_{c+V}(x_i) - c\| - r)^2 \\
&= \sum_{i=1}^{n} \|x_i - c - VV^T(x_i - c)\|^2 + \sum_{i=1}^{n}(\|c + VV^T(x_i - c) - c\| - r)^2 \\
&= \sum_{i=1}^{n} \|x_i - c - VV^T(x_i - c)\|^2 + \sum_{i=1}^{n}(\|VV^T(x_i - c)\| - r)^2
\end{aligned}$$

The second identity comes from the Pythagorean theorem and $Pr_{c+V}(x_i)$ is the linear projection of $x_i$ to the affine subspace $c + V$.

The first sum corresponds to PCA loss function and the second term is the loss of SRCA if $V = I$. For the SRCA and the SPCA, we minimize the first sum so $V$ is the top eigenvectors of sample covariance matrices and then plug this $V$ to the second sum, and change the geometric sum to the algebraic loss function, since only the latter loss allows a closed form analytic solution. This minimizer from a two-step procedure obtained by SPCA is not necessarily the same as the true minimizer of the above geometric loss $\mathscr{L}(V, c, r)$. However, these two minimizers coincide when all $x_i$ are from a sphere, otherwise SPCA solution is sub-optimal (Li et al., 2022). We adopt the two-step SPCA algorithm only because we cannot derive a closed form minimizer for $L = \mathscr{L}(V, c, r)$. Moreover, this loss function is difficult to generalize to the ellipsoid situation.

When the axes of an ellipsoid are parallel to the coordinate axes, it simplifies the problem of finding the distance from any external point to the surface of the ellipsoid. The ellipsoid in $\mathbb{R}^d$ can be represented by the equation:

$$\frac{(x-h)^2}{a^2} + \frac{(y-k)^2}{b^2} + \frac{(z-l)^2}{c^2} = 1$$

where $(h, k, l)$ is the center of the ellipsoid, and $a, b, c$ are the lengths of its semi-axes along the $x, y, z-$axes, respectively.

The closed-form solution for the distance from a point $(x_0, y_0, z_0)$ to the surface of such an ellipsoid is not straightforward and involves solving a system of nonlinear equations. Specifically we can formulate the Lagrangian for the problem, incorporating the constraint (the ellipsoid equation) and the distance function (the Euclidean distance from the point to a variable point on the ellipsoid). In mathematical terms, the Lagrangian $\mathcal{L}$ is:

$$\mathcal{L}(x,y,z,\eta) = \sqrt{(x-x_0)^2 + (y-y_0)^2 + (z-z_0)^2} + \eta\left(\frac{(x-h)^2}{a^2} + \frac{(y-k)^2}{b^2} + \frac{(z-l)^2}{c^2} - 1\right)$$

where $\eta$ is a Lagrange multiplier. The solution involves finding the values of $x, y, z$ and $\eta$ that satisfy the following system of equations derived from the Lagrangian:

$$\frac{\partial \mathcal{L}}{\partial x} = 0, \frac{\partial \mathcal{L}}{\partial y} = 0, \frac{\partial \mathcal{L}}{\partial z} = 0, \frac{\partial \mathcal{L}}{\partial \eta} = 0.$$

Solving these equations yields the closest point on the ellipsoid to the given point, and the distance is the Euclidean distance between these two points. Note that solving this system of equations can be quite complex and may not always lead to a simple closed-form expression, especially for higher-dimensional ellipsoids. In many cases, numerical methods are used to find an approximate solution.

## Appendix H. Related Proofs

### H.1 Proof of Theorem 1

For each fixed $\|v\|_{l_0} = d' + 1$, it suffices to optimize the following sub-problem of (4):

$$\min_{c\in\mathbb{R}^d, r\in\mathbb{R}^+} \sum_{i=1}^{n} \left( (x_i - c)^T W (x_i - c) + r^2 - 2r\sqrt{(x_i - c)^T \sqrt{W}^T v^T I v \sqrt{W}\,(x_i - c)} \right) \tag{12}$$

$$= \min_{c\in\mathbb{R}^d, r\in\mathbb{R}^+} \mathscr{L}_v(c, r; x_1, x_2, \cdots, x_n),$$

$$= \min_{c\in\mathbb{R}^d, r\in\mathbb{R}^+} \sum_{i=1}^{n} \mathscr{L}_v(c, r; x_i), \tag{13}$$

which has gradients with respect to $c$ and $r$ as

$$\begin{aligned}
\frac{\partial \mathscr{L}_v}{\partial c} &= \sum_{i=1}^{n} \frac{\partial \mathscr{L}_v}{\partial c}(c, r; x_i) \\
&= \sum_{i=1}^{n} \Big( -2(x_i - c)^T W - 2r \cdot \frac{1}{2} \left[ (x_i - c)^T \sqrt{W}^T v^T I v \sqrt{W}\,(x_i - c) \right]^{-\frac{1}{2}} \\
&\left[ -2\,(x_i - c)^T \sqrt{W}^T v^T I v \sqrt{W} \right] \Big) \\
&= \sum_{i=1}^{n} - 2(x_i - c)^T \left( W + r \left[ (x_i - c)^T \sqrt{W}^T v^T I v \sqrt{W}\,(x_i - c) \right]^{-\frac{1}{2}} \left[ \sqrt{W}^T v^T I v \sqrt{W} \right] \right),
\end{aligned}$$

and,

$$\frac{\partial \mathscr{L}_v}{\partial r} = \sum_{i=1}^{n} \frac{\partial \mathscr{L}_v}{\partial r}(c, r; x_i) = \sum_{i=1}^{n} \left( 2r - 2\left[ (x_i - c)^T \sqrt{W}^T v^T I v \sqrt{W}\,(x_i - c) \right]^{\frac{1}{2}} \right).$$

Therefore, we can assume that the mild assumptions $\|x_i - c\| \leq R_1, r \leq R_2$ and $|\lambda_{\max}(W)| \leq R_3$. We can compute the bounds of these gradients, using Cauchy-Schwartz

inequality in the first inequality:

$$
\begin{aligned}
\|\nabla_{(c,r)}\mathscr{L}_v(c,r)\| &= \left\|\frac{\partial \mathscr{L}_v}{\partial c}(c,r)\right\| + \left\|\frac{\partial \mathscr{L}_v}{\partial r}(c,r)\right\| \\
&\leq 4\sum_{i=1}^{n}(x_i-c)^T W^T W(x_i-c) \\
&\times \sum_{i=1}^{n}\left\|\left(W + r\left[(x_i-c)^T\sqrt{W}^T v^T I v\sqrt{W}(x_i-c)\right]^{-\frac{1}{2}}\left[\sqrt{W}^T v^T I v\sqrt{W}\right]\right)\right\|^2 \\
&+ \textstyle\sum_{i=1}^{n}\left(2r - 2\left[(x_i-c)^T\sqrt{W}^T v^T I v\sqrt{W}(x_i-c)\right]^{\frac{1}{2}}\right). \\
&\leq 4\times 2nR_3^2 \times nR_1^2 \times n\left(R_3 + R_2\frac{\sqrt{R_3^2}}{\sqrt{R_1^2}}\right) + n\left(2R_2 + \sqrt{R_1^2 R_3^2}\right) \\
&< \infty
\end{aligned}
$$

For a finite $n$, we can conclude that $\mathscr{L}$ is Lipschitz with a finite Lipschitz constant as bounded above. Then the gradient descent algorithm would give us a solution to the sub-problem (13) with linear convergence from classical results (Boyd et al., 2004). Since for fixed $v$, each sub-problem converges to the solution, the exhaustive search on $v$ solves the original problem (4). In parallel to Boyd et al. (2003), we have proved the Theorem 1.

### H.2 Proof of Theorem 2

It is clear that $\mathscr{L}(c_0, r_0, \mathcal{I}_0) = 0$ and $\widehat{\mathcal{I}}_k, \widehat{c}_k, \widehat{r}_k \to \arg\min \mathscr{L}$ by Theorem 1, it suffices to show $(c_0, r_0, \mathcal{I}_0)$ is the unique zero of $L$. Recall that $\mathscr{L}(c, r, \mathcal{I}) = 0$ if and only if all all $x_i$'s are exactly on sphere $S(c, r, \mathcal{I})$, and that $d' + 2$ points uniquely determine a $d'$ dimensional sphere, then the uniqueness follows from the assumption $n > d' + 1$.

### H.3 Proof of Theorem 3

We consider the closed set $\Theta_1$ on the parameter space defined by $\|x_i - c\| \leq R_1,\ \forall i = 1\cdots, n,\ r \leq R_2$ and $|\lambda_{\max}(W)| \leq R_3$ as we did in the proof of Theorem 4 and 5.

Again, let us assume $\mathcal{I}$ to be fixed index set and the

$$
f_\infty(c,r) = \lim_{n\to\infty}\frac{1}{n}\sum_{i=1}^{n}\left((y_i-c)^T W(y_i-c) - r - 2r\sqrt{(y_i-c)^T\sqrt{W}^T I_{\mathcal{I}}\sqrt{W}(y_i-c)}\right)^2
$$

be the limiting form of our geometric loss function,

$$
\mathcal{L}(c,r,\mathcal{I}\mid\mathcal{Y}) = f_j(c,r) = \frac{1}{j}\sum_{i=1}^{j}\left((y_i-c)^T W(y_i-c) - r - 2r\sqrt{(y_i-c)^T\sqrt{W}^T I_{\mathcal{I}}\sqrt{W}(y_i-c)}\right)^2
$$

with the dataset $\mathcal{Y} = \{y_1, \cdots, y_j\}$ and

$$
\mathcal{L}(c,r,\mathcal{I}\mid\mathcal{X}) = g_j(c,r) = \frac{1}{j}\sum_{i=1}^{j}\left((x_i-c)^T W(x_i-c) - r - 2r\sqrt{(x_i-c)^T\sqrt{W}^T I_{\mathcal{I}}\sqrt{W}(x_i-c)}\right)^2
$$

with the dataset $\mathcal{X} = \{x_1, \cdots, x_j\}$. Recall that $x_i = y_i + \epsilon_i$ and $y_i \in S_W(c_0, r_0)$ lying on an ellipsoid with center $c_0$, radius $r_0$ and known covariance $W$.

Therefore, $\arg\min f_{\infty=} \arg\min f_j = (c_0, r_0)$ since if we plug in $c_0$ and $r_0$ the $f_\infty(c_0, r_0) = f_j(c_0, r_0) = 0$. By the definition of $f_\infty$, $f_j$ converges to $f_\infty$ point-wise. In addition, by the compact assumptions, the convergence is also uniform, that is, $\sup_{\theta\in\Theta_1} |f_j(\theta) - f_\infty(\theta)| \to 0$.

The rest of our roadmap of proof is as follows. According to the Remark 1.10 of Braides et al. (2002): if a sequence of functions $g_j$ point-wisely converges to its limit $f_\infty$ uniformly. and $f_\infty$ is lower semi-continous, then the same sequence of functions also converges in a $\Gamma$-convergence sense, and its $\Gamma$-limit is identical to its point-wise limit $f_\infty = \lim_{j\to\infty} f_j$ . Furthermore, as we showed above, the following minimizer exists

$$\theta_* := \arg\min_{\theta\in\Theta_1} f_\infty(\theta).$$

Then since the specific form of our geometric loss function (1) is coercive, by Theorem 1.21 and Remark 1.22 in Braides et al. (2002), the minimizer sequence $\{\theta_j\} = \{\arg\min_{\theta\in\Theta_1} f_j(\theta)\}$ converges to a minimum point $\theta_*$ of $f_\infty$. Note that our assumption (A1) stating that each of $\theta_j := \arg\min_{\theta\in\Theta_1} g_j(\theta)$ exist is essential here. Otherwise the sequence will not exist.

It's it clear that $f_j$ uniformly converges to $f_\infty$ on compact set $\Theta_1$. We focus on proving $g_j$ also converges to $f_j$ uniformly, then through a middle-man argument, $\lim_{j\to\infty} g_j = f_\infty$ holds. The difference between two sequences $f_j$ and $g_j$ can be bounded as below:

$$\begin{aligned}
&|g_j(c, r) - f_j(c, r)| && (14)\\
&= \left| \frac{1}{j} \sum_{i=1}^{j} (y_i - c)^T W (y_i - c) - (x_i - c)^T W (x_i - c) + \right.\\
&\left. 2r\sqrt{(x_i - c)^T \sqrt{W}^T I_{\mathcal{I}} \sqrt{W} (x_i - c)} - 2r\sqrt{(y_i - c)^T \sqrt{W}^T I_{\mathcal{I}} \sqrt{W} (y_i - c)} \right| && (15)\\
&\le \left(4R_3^2 + R_1 R_3\right) \left| \frac{1}{j} \sum_{i=1}^{j} (\|x_i - c\| + \|y_i - c\| - 2r)(\|x_i - c\| - \|y_i - c\|) \right| && (16)\\
&= \left(4R_3^2 + R_1 R_3\right) \left| \frac{1}{j} \sum_{i=1}^{j} (\|x_i - c\| + \|y_i - c\| - 2r)(\|y_i - c + \epsilon_i\| - \|y_i - c\|) \right|\\
&\le \left(4R_3^2 + R_1 R_3\right) \frac{1}{j} \sum_{i=1}^{j} |(\|x_i - c\| + \|y_i - c\| - 2r)|(\|\epsilon_i\| + 2\|\epsilon_i\|\|y_i - c\|) && (17)\\
&= \left(4R_3^2 + R_1 R_3\right) \frac{1}{j} \sum_{i=1}^{j} |(\|x_i - c\| + \|y_i - c\| - 2r)|(\|\epsilon_i\| + 2\|y_i - c\|) \cdot \|\epsilon_i\|\\
&\le \left(4R_3^2 + R_1 R_3\right) \cdot (2R_1 + 2R_2) \cdot (1 + 2R_1) \cdot \frac{1}{j} \sum_{i=1}^{j} \|\epsilon_j\|, && (18)
\end{aligned}$$

where we need the assumption (A2) stating that $x_i, y_i$ and $c, r$ are all in the closed bounded (hence compact) set $B \times \Theta_1$. We want to show that as $j \to \infty$,

$$\sup_{\theta=(c,r)\in\Theta_1} \|g_j - f_j\| \to 0,$$

to ensure uniform convergence. But this follows from (18) and our assumption (A3) stating that $\lim_{n\to\infty}\frac{1}{n}\sum_{i=1}^{n}\|\epsilon_i\|=0$. Such an assumption is common, see, Maggioni et al. (2016); Fefferman et al. (2018); Aamari and Levrard (2019) for instances. In fact, the assumption is even weaker than those in the above references, for example, in Aamari and Levrard (2019) the amplitude of the noise is assume to be $\|\epsilon\|\sim n^{-\frac{\alpha}{d}}$ for $\alpha>1$. In contrast, we only require $\|\epsilon\|\to 0$, so $\|\epsilon\|\sim n^{-\alpha}$ for any $\alpha>0$ or even $\|\epsilon\|\sim\frac{1}{\log n}$ is good enough.

### H.4 Proof of Theorem 4

References we mainly need for our proof below are the formulation in Huber (2004); Huber et al. (1967) and the technical separation lemma in Doob (1953).

We fix the index set $\mathcal{I}$ in the following discussions, and we assume that the parameters to be estimated can be written as a vector $\theta=(c,r)\in\Theta:=[-C,C]^d\times[R_0,R]\subset\mathbb{R}^d\times\mathbb{R}^+$, which lies in a (locally) compact space with a countable base $\Theta'=\left\{[-C,C]^d\cap\mathbb{Q}^d\right\}\times\{[R_0,R]\cap\mathbb{Q}\}$, the inclusion of $r=R_0$ is needed below for compactness. We denote that estimate for $\theta$ based on $n$ samples (by minimization of the $\mathscr{L}$) by $T_n=T_n(\mathcal{X})$.

The real-valued $\rho$ function, based on the samples $x_1,\cdots,x_n\in\mathbb{X}=\mathbb{R}^d$ drawn from the common distribution $P$ defined on the probability space $(\mathbb{X},\mathcal{A},\nu)$ with Borel algebra $\mathcal{A}$ and Lebesgue measure $\nu$, is

$$\rho(x;\theta)=\left((x-c)^TW(x-c)+r^2-2r\sqrt{(x-c)^T\sqrt{W}^TI_{\mathcal{I}}\sqrt{W}\,(x-c)}\right)$$

and the $\psi(x;\theta)=\frac{\partial}{\partial\theta}\rho(x;\theta)$ is again differentiable. We show below that the assumptions in Huber et al. (1967) are satisfied, we define our estimator $T_n$ for parameter $\theta=(c,r)$ such that

$$\frac{1}{n}\sum_{i=1}^{n}\rho(x_i;T_n)-\inf_{\theta\in\Theta}\frac{1}{n}\sum_{i=1}^{n}\rho(x_i;\theta)\to 0,\text{a.s. } P\qquad\text{when } n\to\infty,$$

corresponding to case A in Huber et al. (1967). Since $\rho$ is differentiable in both $x,\theta$, this minimizer could also be expressed in form of $T_n$ satisfying

$$\frac{1}{\sqrt{n}}\sum_{i=1}^{n}\psi(x_i;T_n)\to 0,\text{a.s. } P\qquad\text{when } n\to\infty,$$

- (A-1) For $\Theta=\mathbb{R}^d\times\mathbb{R}^+$, there exists a countable basis $\Theta'=\left\{[-C,C]^d\cap\mathbb{Q}^d\right\}\times\{[R_0,R]\cap\mathbb{Q}\}$ such that for every open set $U\subset\Theta$ and every closed interval $A\subset\mathbb{R}$, two sets

$$\{x\mid\rho(x;\theta)\in A\in\mathcal{A},\forall\theta\in U\}$$
$$\{x\mid\rho(x;\theta)\in A\in\mathcal{A},\forall\theta\in U\cap\Theta'\}$$

would only differ on the set of zero probability measure $P$. Since the measure $P$ is fixed, by Lemma 2.1 on page 56 of Doob (1953), for each $\theta\in\Theta$ we can find $\theta'\in\Theta'$ such that

$$P\left\{\omega\in\mathbb{X}\mid\rho(x(\omega);\theta)\neq\rho(x(\omega);\theta'),x(\omega)\sim P\right\}=0.$$

Therefore, denote the map $\tau_P : \theta \mapsto \theta'$ we can redefine our $\rho$ by $\tilde{\rho} := \rho \circ \tau_P$ so that it only differs from $\rho$ on a zero measure set of the fixed $P$. Note that the mapping $\tau_P$ depends on the measure $P$ and we assume $P$ is fixed throughout our discussion. This ensures the measurability of $\inf_{\theta' \in U} \tilde{\rho}(x; \theta')$ and the measurability of its limit when an (open) neighbor hood $U$ of $\theta$ shrinks to one-point set $\{\theta\}$. For ease of notation, we still use $\rho$ below as assume (A-1) holds.

- (A-2) The function $\rho$ is continuous and differentiable, therefore clearly lower semi-continuous in $\theta = (c, r)$. And this ensures that $\inf_{\theta' \in U} \rho(x; \theta') \to \rho(x; \theta)$.

- (A-3) There exists a measurable function $a(x)$ such that

$$\mathbb{E}_P \left(\rho(x; \theta) - a(x)\right)^- < \infty$$
$$\mathbb{E}_P \left(\rho(x; \theta) - a(x)\right)^+ < \infty$$

and hence $\gamma(\theta) = \mathbb{E}\left(\rho(x, \theta) - a(x)\right)$ is well-defined for all $\theta \in \Theta$. For our purpose, we choose $\theta_1 = (c_1, r_1)$ for some $\|c_1\| < \infty$ and $r_1 < \infty$. We define a function on $\mathbb{X}$

$$\begin{aligned} a(x) = a_{\theta_1}(x) &= \left( (x - c_1)^T W (x - c_1) + r_1^2 - 2 \cdot r_1 \sqrt{(x - c_1)^T \sqrt{W}^T I_{\mathcal{I}} \sqrt{W} (x - c_1)} \right) \\ \rho(x; \theta) - a(x) &= \left( (x - c)^T W (x - c) - (x - c_1)^T W (x - c_1) \right) + \left( r^2 - r_1^2 \right) \\ &\quad - 2r \sqrt{(x - c)^T \sqrt{W}^T I_{\mathcal{I}} \sqrt{W} (x - c)} \\ &\quad + 2r_1 \sqrt{(x - c_1)^T \sqrt{W}^T I_{\mathcal{I}} \sqrt{W} (x - c_1)} \\ &\leq |\lambda_{\max}(W)| \left( \|x - c\|^2 - \|x - c_1\|^2 \right) + \left( r^2 - r_1^2 \right) \\ &\quad + 4 \max(r, r_1) \cdot \max(\|c\|, \|c_1\|) \cdot |\lambda_{\max}(W)| \cdot \|x\| \end{aligned}$$

If we take $\mathbb{E}_P$ on both sides of inequality above and with the assumption that $|\lambda_{\max}(W)| < R_3$, then the mild assumption that $P$ has finite second moments (hence finite first moment) ensures the finiteness. It is not hard to see that the choice of $\theta_1$ is not essential in verifying this assumption. For simplicity, we assume $\theta_1 = (c_1, r_1) = (0, 1)$ hereafter.

$$a(x) = \left( |\lambda_{\max}(W)| \|x\|^2 + 1 - 2 \sqrt{x^T \sqrt{W}^T I_{\mathcal{I}} \sqrt{W} x} \right)$$

- (A-4) There is a $\theta_0 \in \Theta$ such that $\gamma(\theta) > \gamma(\theta_0)$ for all $\theta \neq \theta_0$. To see this, we use the Fubini theorem to take differentiation inside the $\mathbb{E}_P$ (note that this is taken with respect to $x$) to conclude unique minima of $\gamma(\theta)$ (notice that we assume $\mathcal{I}$ fixed and

therefore the index vector $v$ is a fixed constant vector)

$$\begin{aligned}
\gamma(\theta) &= \mathbb{E}_P\rho(x;\theta) - a(x) \\
&= \mathbb{E}_P\left((x-c)^T W(x-c) + r^2 - 2r\sqrt{(x-c)^T\sqrt{W}^T I_{\mathcal{I}}\sqrt{W}\,(x-c)}\right) - a(x) \\
\frac{\partial}{\partial\theta}\gamma(\theta) &= \mathbb{E}_P\begin{pmatrix} \frac{\partial}{\partial c}\rho(x;\theta) \\ \frac{\partial}{\partial r}\rho(x;\theta)\end{pmatrix} \\
&= \begin{pmatrix} -\mathbb{E}_P 2(x-c)^T\left(W + r\left[(x-c)^T\sqrt{W}^T v^T I_p v\sqrt{W}\,(x-c)\right]^{-\frac{1}{2}}\left[\sqrt{W}^T v^T I_p v\sqrt{W}\right]\right) \\ \mathbb{E}_P 2r - 2\left[(x-c)^T\sqrt{W}^T v^T I_p v\sqrt{W}\,(x-c)\right]^{\frac{1}{2}}\end{pmatrix} \\
&= 0
\end{aligned}$$

By letting $\frac{\partial}{\partial\theta}\gamma(\theta) = 0$ and for $x \sim P$, we derive from the second equation that

$$r_0(c) = \mathbb{E}_P\left[(x-c)^T\sqrt{W}^T v^T I_p v\sqrt{W}\,(x-c)\right]^{\frac{1}{2}} \in [0, \min(R, 2C\sqrt{|\lambda_{\max}(W)|})],$$

and from the first equation

$$\mathbb{E}_P 2\,(x-c)^T\left(W + r_0(c)\left[(x-c)^T\sqrt{W}^T v^T I_p v\sqrt{W}\,(x-c)\right]^{-\frac{1}{2}}\left[\sqrt{W}^T v^T I_p v\sqrt{W}\right]\right) = 0$$

Consider the following function

$$F(c) \coloneqq \mathbb{E}_P 2\,(x-c)^T\left(W + r_0(c)\left[(x-c)^T\sqrt{W}^T v^T I_p v\sqrt{W}\,(x-c)\right]^{-\frac{1}{2}}\left[\sqrt{W}^T v^T I_p v\sqrt{W}\right]\right) \quad (19)$$

as a function of $c$ and the above equation becomes $F(c) = 0$. Taking a sandwiching-style argument, we first note that the second term in the second bracket is always non-negative, then we construct uniform bounding functions:

$$\begin{aligned}
F_1(c) &\coloneqq \mathbb{E}_P 2\,(x-c)^T W \\
&\asymp \mathbb{E}_P\,(x-c)^T W, \\
F_2(c) &\coloneqq \mathbb{E}_P 2\,(x-c)^T\Big(W + \min(R, 2C\sqrt{|\lambda_{\max}(W)|})\cdot \\
&\quad |\lambda_{\max}(W)|\left[(x-c)^T\sqrt{W}^T v^T I_p v\sqrt{W}\,(x-c)\right]^{-\frac{1}{2}}\Big) W \\
&\asymp \mathbb{E}_P\,(x-c)^T\left(1 + \frac{K(R, C, v, |\lambda_{\max}(W)|)}{\|x-c\|_2}\right) W,
\end{aligned}$$

(where $K(R, C, v, |\lambda_{\max}(W)|)$ is a non-negative constant) such that the following bound $F_1(c) \leq F(c) \leq F_2(c)$ holds (for each component of the vector-valued $F_1, F_2$) uniformly in $c$. However, it is clear that there exists $c_1^+, c_2^- \in [-C, C]^d \subset \mathbb{R}^d$

$$\begin{aligned}
F(c_1^+) &\geq F_1(c_1^+) > 0, \\
F(c_2^-) &\leq F_2(c_2^-) < 0.
\end{aligned}$$

Note that $F$ is continuous in $c$ (we can take derivative under $\mathbb{E}_P$ since $P$ is assumed to possess finite second moment) and $[-C, C]^d$ is connected, we apply the multivariate intermediate value theorem to assert the existence of **a** solution $c_0$ for $F(c) = 0$. Therefore, we can keep this solution $c_0$, which we know its existence but do not know its expression. Back substitution of this solution of $c_0$ into the expression of $r_0$ yields

$$r_0 = \mathbb{E}_P \left[ (x - c_0)^T \sqrt{W}^T v^T I_p v \sqrt{W} (x - c_0) \right]^{\frac{1}{2}},$$

where $\theta_0 = (c_0, r_0)$ is well-defined for $P$ with finite second moment. This verifies the assumption (A-4).

- (A-5) With the notations in (A-3), since $\Theta := [-C, C]^d \times [R_0, R] \subset \mathbb{R}^d \times \mathbb{R}^+$ is a compact space, it suffices to verify only (i) of (A-5). There is a continuous function $b(\theta) > 0$ such that

$$b(\theta) = \left( c^T W c + r^2 - 2r \sqrt{c^T \sqrt{W}^T I_{\mathcal{I}} \sqrt{W} c} \right) + 1 \in [1, 1 + C^2 |\lambda_{\max}(W)| + R^2]$$

For a fixed $x \in \mathbb{X}$, the function $g_x(\theta) = 2r \sqrt{(x - c)^T \sqrt{W}^T I_{\mathcal{I}} \sqrt{W} (x - c)}$ has gradient

$$\begin{aligned} \frac{\partial}{\partial \theta} g_x(c, r) &= \begin{pmatrix} \frac{\partial}{\partial c} g_x(c, r) \\ \frac{\partial}{\partial r} g_x(c, r) \end{pmatrix} \\ &= \begin{pmatrix} r \left[ (x - c)^T \sqrt{W}^T I_{\mathcal{I}} \sqrt{W} (x - c) \right]^{-\frac{1}{2}} \cdot 2 (x - c)^T \sqrt{W}^T I_{\mathcal{I}} \sqrt{W} \\ 2 \sqrt{(x - c)^T \sqrt{W}^T I_{\mathcal{I}} \sqrt{W} (x - c)} \end{pmatrix} \end{aligned}$$

which is bounded from above in matrix norm by $2R\sqrt{|\lambda_{\max}(W)|} \cdot 4RC\sqrt{|\lambda_{\max}(W)|} \leq 16 \max(R^2, 1) \cdot C|\lambda_{\max}(W)| =: L_g < \infty$. Therefore, the function $g_x(c, r)$ is a $L_g$-Lipschitz function. We have

$$\begin{aligned} \inf_{\theta \in \Theta} \frac{\rho(x; \theta) - a(x)}{b(\theta)} &= \inf_{\theta \in \Theta} \Big\{ \big( (x - c)^T W (x - c) - x^T W x \big) + \big( r^2 - 1 \big) \\ &\quad - 2r \sqrt{(x - c)^T \sqrt{W}^T I_{\mathcal{I}} \sqrt{W} (x - c)} + 2 \sqrt{x^T \sqrt{W}^T I_{\mathcal{I}} \sqrt{W} x} \Big\} \\ &\quad \left( c^T W c + r^2 - 2r \sqrt{c^T \sqrt{W}^T I_{\mathcal{I}} \sqrt{W} c} + 1 \right)^{-1} \\ &\geq \inf_{\theta = (c, r) \in \Theta} \Big\{ \big( (x - c)^T W (x - c) - x^T W x \big) + \big( r^2 - 1 \big) \\ &\quad - 2r \sqrt{(x - c)^T \sqrt{W}^T I_{\mathcal{I}} \sqrt{W} (x - c)} + 2 \sqrt{x^T \sqrt{W}^T I_{\mathcal{I}} \sqrt{W} x} \Big\} \\ &\quad (1 + C^2 |\lambda_{\max}(W)| + R^2)^{-1} =: h(x) \end{aligned}$$

and $\frac{\rho(x, \theta) - a(x)}{b(\theta)} \geq h(x)$ by the infimum in the definition while $h(x)$ is integrable with respect to $P$ due to the fact that $g_x(c, r)$ is Lipschitz.

Now we verify all assumptions (A-1) to (A-5) in Huber et al. (1967), Theorem 1 in the same paper ensures that Theorem A holds. The mild assumptions that $\theta$ lies in a compact subspace of $\mathbb{R}^d \times \mathbb{R}^+$ can be relaxed by verifying a more stringent set of conditions (A-5) as pointed out by Huber et al. (1967). Since we actually verify assuming that the index set $\mathcal{I}$ is fixed, we need to point out that in the $l_0$ optimization for each fixed $\mathcal{I}$ the consistency result holds. But for the $l_1$ relaxed problem, we cannot guarantee consistency even with stronger assumptions, only algorithmic convergence is guaranteed.

### H.5 Proof of Theorem 5

The key idea of our proof is that we can treat our estimators $T_n$ as the solution of a robust estimation problem for the parameters $(c, r)$ if the index $\mathcal{I}$ is fixed. Now we take the second view that the estimator sequence $T_n$ for parameter $\theta = (c, r)$ and assume a fixed index set $\mathcal{I}$ such that

$$\frac{1}{n}\sum_{i=1}^{n} \psi(x_i; T_n) \to 0, \text{a.s. } P$$

$$n \to \infty,$$

- (N-1) For each fixed $\theta \in \Theta$, $\psi(x;\theta)$ is $\mathcal{A}$-measurable and separable. Like the construction in (A-1), we can modify the $\psi$ into a separable version $\tilde{\psi}$ if necessary and verify this assumption. Then following functions are well-defined (with finite second moment assumption on $P$ and the Fubini theorem)

$$\begin{aligned}
\lambda(\theta) = \lambda(c, r) &:= \mathbb{E}_P \psi(x;\theta) \\
&= \mathbb{E}_P \frac{\partial}{\partial\theta}\rho(x;\theta) \\
&= \frac{\partial}{\partial\theta}\mathbb{E}_P \rho(x;\theta) \\
u(x, \theta, D) &= \sup_{\|\tau-\theta\|\le D} |\psi(x;\tau) - \psi(x;\theta)| .
\end{aligned}$$

- (N-2) The same $\theta_0$ as computed above would satisfy $\lambda(\theta_0) = 0$.

- (N-3) There are strictly positive numbers $\alpha, \beta, \gamma, \eta$ such that

  - (i) $|\lambda(\theta)| \ge \alpha|\theta - \theta_0|$ for some $\alpha > 0$ and $|\theta - \theta_0| \le \eta$ is clear since

$$\lambda(\theta) = \frac{\partial}{\partial\theta}\mathbb{E}_P \left( (x-c)^T W (x-c) + r^2 - 2r\sqrt{(x-c)^T \sqrt{W}^T I_{\mathcal{I}} \sqrt{W}\,(x-c)} \right)$$

    is quadratic in both $c$ and $r$, and it is bounded from below by linear part due to Taylor expansion at $\theta_0$.

  - (ii) $\mathbb{E}_P u(x,\theta,D) = \mathbb{E}_P \sup_{\|\tau-\theta\|\le D} |\psi(x;\tau) - \psi(x;\theta)| \le \mathbb{E}_P \beta\|\tau-\theta\|$ since $\frac{\partial}{\partial r}\psi(x;\theta) = 2$ and

$$\frac{\partial}{\partial c}\psi(x;\theta) = \mathbb{E}_P 2c^T \left( W + r \left[ (x-c)^T \sqrt{W}^T v^T I v \sqrt{W} (x-c) \right]^{-\frac{1}{2}} \left[ \sqrt{W}^T v^T I v \sqrt{W} \right] \right)$$
$$- \mathbb{E}_P 2(x-c)^T (-\frac{1}{2} r \left[ (x-c)^T \sqrt{W}^T v^T I v \sqrt{W} (x-c) \right]^{-\frac{3}{2}}$$
$$\cdot 2 (x-c)^T \sqrt{W}^T v^T I v \sqrt{W} \cdot \left[ \sqrt{W}^T v^T I v \sqrt{W} \right])$$
$$\leq 2CR(1 + (4C^2 |\lambda_{\max}(W)|^{-\frac{1}{2}}) |\lambda_{\max}(W)|$$
$$+ 4C \left( R \cdot (4C^2 |\lambda_{\max}(W)|)^{-\frac{3}{2}} \cdot 2C |\lambda_{\max}(W)|^2 \right)$$
$$\leq 16CR(4C^2 |\lambda_{\max}(W)|^{-\frac{1}{2}} \max(|\lambda_{\max}(W)|, 1)^4 + |\lambda_{\max}(W)| < \infty.$$

And $\psi(x;\theta)$ is Lipschitz with coefficient

$$\beta := 32CR(4C^2 |\lambda_{\max}(W)|^{-\frac{1}{2}} \max(|\lambda_{\max}(W)|, 1)^4 + |\lambda_{\max}(W)|.$$

– (iii) $\mathbb{E}_P u(x,\theta,D)^2 = \mathbb{E}_P \left( \sup_{\|\tau-\theta\|\leq D} |\psi(x;\tau) - \psi(x;\theta)| \right)^2 \leq \max \left\{ \mathbb{E}_P \left( \beta \|\tau-\theta\| \right)^2, \left( \mathbb{E}_P \beta \|\tau-\theta\| \right)^2 \right\}$ and for $\gamma = \beta$ we can replace $\|\tau-\theta\| \leq \eta - D$ with $\eta - D$.

- (N-4) $\mathbb{E}_P \left[ |\psi(x;\theta_0)|^2 \right] < \infty$ is clear from the analytic expression of $\psi(x;\theta)$, which involves at most quadratic entries in $x$, and the fact that we assume $P$ has finite second moments.

Assumptions (N-1) through (N-4) allow us to apply Theorem 3 and its corollary in Huber et al. (1967) and claim Theorem B.

The asymptotic normality result allows us to claim a Wald-type hypothesis testing for the estimated center and radius for the sphere for a fixed index set $\mathcal{I}$. that aspect in the current paper but point out that this is one of the few non-bootstrap hypothesis testing methods in manifold learning literature.

### H.6 Proof of Theorem 6

First we compare SRCA with PCA. Assume $\|x_i\| \leq \alpha$ for any $i$, then for any $\epsilon > 0$, there exists a sphere $S_\epsilon$ such that $d(y, S_\epsilon) \leq \epsilon$ for any $y \in H$ with $\|y\| \leq \alpha$ (Li et al., 2022). Intuitively, a plane can be approximated by a sphere with infinite radius. Let $\widehat{x}_i = \arg\min_{y\in H} d(x_i, y)$ be the linear projection of $x_i$ to plane $H$, then by the triangle inequality,

$$d(x_i, S_\epsilon) \leq d(x_i, \widehat{x}_i) + d(\widehat{x}_i, S_\epsilon).$$

Since the linear projection of a bounded set is still bounded, $d(\widehat{x}_i, S_\epsilon) \leq \epsilon$. By the definition of SRCA,

$$\sum_{i=1}^n d^2(x_i, S_2) \leq \sum_{i=1}^n d^2(x_i, S_\epsilon) \leq \sum_{i=1}^n d^2(x_i, H) + 2\epsilon \sum_{i=1}^n d(x_i, H) + n\epsilon^2.$$

Let $\epsilon \to 0$, we conclude that

$$\sum_{i=1}^{n} d^2(x_i, S_2) \leq \sum_{i=1}^{n} d^2(x_i, H).$$

Then we compare SRCA with SPCA. Since the objective function $\mathscr{L}$, which defines SRCA, is $\min_S \sum_{i=1}^{n} d^2(x_i, S)$, it follows from $S_1 \subset H$ that

$$\sum_{i=1}^{n} d^2(x_i, S_2) \leq \sum_{i=1}^{n} d^2(x_i, S_1).$$

Note that SPCA is a restricted version of SRCA, where $\mathcal{I} = \{1, \cdots, d' + 1\}$.

## Appendix I. Mean Square Errors for Out-of-sample Data

This section provides the out-of-sample mean square errors of PCA, SPCA and SRCA on the same datasets in Table 2. We provide this to show that performance evaluation measures are not really affected by the choice of testing samples.

## Appendix J. Branch-and-bound

We provided a supplementary algorithm, namely the branch-and-bound (BnB) implementation for solving the binary search problem originally treated in Algorithm 1. The branch-and-bound algorithm (Lawler and Wood, 1966; Morrison et al., 2016) introduces the branching structure for searching space, and takes an additional tolerance parameter $\tau$ to trade the loss of precision with reduced complexity for mixed integer problems. However, to avoid introducing more parameters, we can use the following slightly different implementation of branch-and-bound for a fixed target reduced dimension $d'$ to reduce the number of function evaluations in solving the SRCA problem (See Algorithm 3). This algorithms searches for an exact, rather than approximate, solution to the optimization problem. It will continue branching and evaluating until it either finds the optimal solution or has considered all possibilities.

Branching: The algorithm generates branches in the solution space by including or excluding dimensions (features) from the current subset $\mathcal{I}_{cur}$. This is done by maintaining a queue of selections to explore. For each selection, the algorithm determines the next dimension to consider and creates a new branch by including that dimension. This branch is then added to the queue to be explored later.

Bounding: At each step, the algorithm calculates a loss value $\mathscr{L}$ based on a loss function that measures the quality of the current selection of features. If this value is lower than the best found so far, the algorithm updates the best solution. This step acts as a bound, because it allows the algorithm to discard branches that cannot possibly be better than the current best solution based on the loss function's value. In other words, if a partial solution has a worse loss than the current best, further exploration of that branch can be stopped.

Pruning: The condition that the number of selected dimensions must match target dimension $d'$ ensures that only feasible solutions are evaluated. This prunes the search space by avoiding the evaluation of incomplete or oversized feature sets.

| Dataset | Method/$d'$ = | 1 | 2 | 3 | 4 |
|---|---|---|---|---|---|
| Banknote | PCA | 15.6094 | 6.2737 | 1.9278 | |
| | SPCA | 15.0516 | 7.3182 | 1.5511 | |
| | SRCA | **13.2273** | **5.4120** | **1.1257** | |
| Power Plant | PCA | 227.6291 | 56.0524 | 23.4302 | **3.0244** |
| | SPCA | 151.7555 | 102.3262 | 44.3802 | 41.0081 |
| | SRCA | **151.3426** | **52.9769** | **20.0871** | 4.0775 |
| User Knowledge | PCA | 0.1952 | 0.1281 | 0.0749 | 0.0306 |
| | SPCA | 0.1478 | 0.0898 | 0.0465 | 0.0145 |
| | SRCA | **0.1479** | **0.0904** | **0.0462** | **0.0144** |
| Ecoli | PCA | 0.0761 | 0.0334 | 0.0219 | **0.0057** |
| | SPCA | **0.0462** | 0.0351 | 0.0187 | 0.0122 |
| | SRCA | 0.0758 | **0.0337** | **0.0168** | 0.0058 |
| Concrete | PCA | 6.8783 | 4.8345 | 3.5462 | 2.5046 |
| | SPCA | **5.5565** | **4.2051** | **3.1664** | **2.0173** |
| | SRCA | 5.5573 | 4.2173 | 3.1842 | 2.0389 |
| Leaf | PCA | 0.0245 | 0.0126 | **0.0062** | 0.0040 |
| | SPCA | **0.0163** | **0.0100** | 0.0073 | 0.0047 |
| | SRCA | 0.0164 | 0.0101 | **0.0062** | **0.0037** |
| Climate | PCA | 1.4486 | 1.3846 | 1.3167 | 1.2447 |
| | SPCA | 1.4265 | 1.3637 | 1.2921 | 1.2224 |
| | SRCA | **1.3863** | **1.3081** | **1.2278** | **1.1525** |

Table 17: Out-of-sample mean square error (MSE) table for different experiments.

The algorithm proceeds by exploring the search space in a breadth-first manner (though it could be adapted to depth-first or best-first), evaluating potential solutions, and pruning the search tree based on the loss function's values. The combination of these branching, bounding, and pruning strategies defines the branch-and-bound nature of the algorithm.

From Tabel 18, Binary search (BS) demonstrates a superior ability to minimize Mean Squared Error (MSE). For the Banknote dataset, characterized by its lower-dimensional space, BS excels, improving as more dimensions are considered. This trend suggests that BS is particularly effective in straightforward scenarios where precision is essential.

In the context of User Knowledge datasets, which present more complexity and benefit from a higher-dimensional feature space, BS continues to outperform other methods. Despite an initial increase in MSE observed with $l_1$ relaxation in the Power Plant dataset,

BS maintains the smallest MSE across all dimensions, showcasing its adept handling of multifaceted structures.

With the Ecoli dataset, where the differences in performance between methods are less pronounced, BS still manages to achieve a marginally lower MSE, further reinforcing its applicability even in subtle and nuanced datasets.

In the Power Plant dataset, BS shows a significantly higher count of evaluations compared to $l_1$ and Branch-and-Bound (BnB), which may indicate a trade-off between its precision in MSE and computational efficiency. With BS requiring more evaluations as dimensionality grows, underscoring its comprehensive search at the expense of computational simplicity.

Conversely, BnB and $l_1$ consistently require fewer evaluations across most datasets and dimensions, signaling their efficiency. Notably, in the Ecoli dataset, the number of evaluations across all methods does not significantly diverge, suggesting that in certain data contexts, the choice of method may be less consequential to computational load.

Overall, while BS stands as a methodological pillar for accuracy in terms of MSE, it comes at the cost of higher computational complexity. In contrast, BnB and $l_1$ demonstrate a more efficient approach, requiring fewer evaluations to achieve their results, which could be preferable in resource-constrained scenarios or when a balance between precision and efficiency is desired. For BnB, it requires careful choice of tolerance parameter, which may serve as a supplement to the $l_1$ approach for very high-dimensional datasets.

**Algorithm 3:** SRCA dimension reduction algorithm with branch-and-bound

**Data:** $X$ (data matrix consisting of $n$ samples in $\mathbb{R}^d$)
**Input:** $d'$ (the dimension of the sphere), $W$ (the covariance weight matrix, by default $W = I_d$), rotationMethod (the method we use to construct the rotation matrix).
**Result:** $\hat{c}$ (The estimated center of $S_{\mathcal{I}}$ in $\mathbb{R}^d$), $\hat{r}$ (The estimated radius of $S_{\mathcal{I}}$), $\mathcal{I}_{opt}$ (The optimal index subset)

`GetRotation` ($X$,rotationMethod) and `ProjectToSphere` ($X$,$c$,$r$,$k$) are identically defined in Algorithm 1.

**begin**
- Standardize the dataset by subtracting its empirical mean $X = X - \bar{X}$
- Construct a rotate matrix $R$ = `GetRotation` ($X$,rotationMethod)
- $X_{rotated} = X * R$, $\mathscr{L}_{opt} = \infty$
- queue $=\{zeros(1, d)\}$.
- **while** *queue is not empty* **do**
  - $\mathcal{I} = \mathcal{I}_{cur}$ popped from queue.
  - **if** $|\mathcal{I}_{cur}|$==$d'$ **then**
    - Solve the optimization problem (2) with respect to $c, r$ with a fixed $\mathcal{I}_{cur}$.
    - Denote the solution as $c_{cur}, r_{cur}, \mathcal{I}_{cur}$
    - **if** $\mathscr{L}(c, r, \mathcal{I} \mid \mathcal{X}) \leq \mathscr{L}_{opt}$ **then**
      - $\mathscr{L}_{opt} \leftarrow \mathscr{L}(c, r, \mathcal{I} \mid \mathcal{X})$
      - $c_{opt} \leftarrow c_{cur}$, $r_{opt} \leftarrow r_{cur}$, $\mathcal{I}_{opt} \leftarrow \mathcal{I}_{cur}$
  - **else**
    - next_dim = find($\mathcal{I}_{cur}$ == 0, 1);
    - `/* find the index of the first occurrence where current selection vector equals 0 */`
    - include_branch = $\mathcal{I}_{cur}$
    - include_branch(next_dim) = 1;
    - queue{end + 1} = include_branch;
  - **end**
- **end**
- Construct the binary index vector $\eta = (\eta_i), \eta_i = 1$ iff $i \in \mathcal{I}$ and $\eta_i = 0$ otherwise.
- $\hat{c} = c_{opt} \cdot \eta * R^{-1} + \bar{X}$, $\hat{r} = r_{opt}$
- $X_{rotated}(:, \mathcal{I}) \leftarrow 0$
- $X_{rotated} \leftarrow$ `ProjectToSphere` ($X$,$\hat{c}$,$\hat{r}$,$k$)
- $X_{output} \leftarrow X_{rotated} * R^{-1} + \bar{X}$

**end**

| Dataset | Method | Metrics | $\mathbf{d'=1}$ | $\mathbf{d'=2}$ | $\mathbf{d'=3}$ | $\mathbf{d'=4}$ |
|---|---|---|---|---|---|---|
| Banknote | BS | # Evaluations | 1254 | 1426 | 409 | |
| | | MSE | 13.439 | 5.5088 | 1.0743 | |
| | $l_1$ | # Evaluations | 793 | 837 | 1097 | |
| | | MSE | 13.439 | 5.5088 | 1.0743 | |
| | BnB | # Evaluations | 168 | 336 | 817 | |
| | | MSE | 13.439 | 5.5088 | 1.0743 | |
| Power Plant | BS | # Evaluations | 4581 | 8886 | 3855 | 1009 |
| | | MSE | 150.8041 | 52.1439 | 19.8839 | 3.3868 |
| | $l_1$ | # Evaluations | 900 | 1779 | 1654 | 4141 |
| | | MSE | 150.8041 | 53.7488 | 19.8839 | 3.3868 |
| | BnB | # Evaluations | 217 | 588 | 581 | 2017 |
| | | MSE | 150.8041 | 53.7488 | 19.8839 | 3.3868 |
| User Knowledge | BS | # Evaluations | 4049 | 5463 | 3134 | 778 |
| | | MSE | 0.14584 | 0.088711 | 0.047064 | 0.014162 |
| | $l_1$ | # Evaluations | 1692 | 2079 | 2285 | 2927 |
| | | MSE | 0.14584 | 0.088711 | 0.047064 | 0.014162 |
| | BnB | # Evaluations | 378 | 560 | 581 | 1555 |
| | | MSE | 0.14584 | 0.088711 | 0.047064 | 0.014162 |
| Ecoli | BS | # Evaluations | 11694 | 27107 | 35009 | 27408 |
| | | MSE | 0.076661 | 0.032799 | 0.018332 | 0.0075651 |
| | $l_1$ | # Evaluations | 2518 | 3000 | 4702 | 3945 |
| | | MSE | 0.076817 | 0.032799 | 0.018332 | 0.0076073 |
| | BnB | # Evaluations | 540 | 720 | 1008 | 1044 |
| | | MSE | 0.076817 | 0.032799 | 0.018332 | 0.0076073 |
| Concrete | BS | # Evaluations | 15942 | 50519 | 97025 | 104791 |
| | | MSE | 5.219 | 3.4745 | 2.1726 | 0.98616 |
| | $l_1$ | # Evaluations | 3054 | 3377 | 2804 | 2747 |
| | | MSE | 5.219 | 3.4745 | 2.1726 | 0.98616 |
| | BnB | # Evaluations | 297 | 407 | 407 | 616 |
| | | MSE | 5.219 | 3.4745 | 2.1726 | 0.98616 |
| Leaf | BS | # Evaluations | 75883 | 411340 | 1334281 | 2951906 |
| | | MSE | 5.2223 | 3.1599 | 1.8433 | 1.1025 |
| | $l_1$ | # Evaluations | 7250 | 6655 | 12812 | 6792 |
| | | MSE | 5.2223 | 3.1599 | 1.9634 | 1.1025 |
| | BnB | # Evaluations | 464 | 464 | 768 | 912 |
| | | MSE | 5.2223 | 3.1599 | 1.9634 | 1.1025 |
| Climate | BS | # Evaluations | 146873 | 845856 | 3464180 | 10939128 |
| | | MSE | 1.3554 | 1.2646 | 1.178 | 1.0905 |
| | $l_1$ | # Evaluations | 17495 | 16748 | 16541 | 15065 |
| | | MSE | 1.3557 | 1.2646 | 1.178 | 1.0905 |
| | BnB | # Evaluations | 720 | 960 | 1160 | 1260 |
| | | MSE | 1.3557 | 1.2646 | 1.178 | 1.0905 |

Table 18: Comparison of different methods of SRCA (BS: binary search in Algorithm 1; $l_1$: $l_1$ relaxation in Algorithm 2; BnB: Branch-and-bound in Algorithm 3 ) across datasets
